# PiDeeL: metabolic pathway-informed deep learning model for survival analysis and pathological classification of gliomas

**DOI:** 10.1093/bioinformatics/btad684

**Published:** 2023-11-11

**Authors:** Gun Kaynar, Doruk Cakmakci, Caroline Bund, Julien Todeschi, Izzie Jacques Namer, A Ercument Cicek

**Affiliations:** Computer Engineering Department, Bilkent University, 06800 Ankara, Turkey; School of Computer Science, McGill University, Montreal, QC, H3A 0E9, Canada; MNMS Platform, University Hospitals of Strasbourg, Strasbourg 67098, France; ICube, University of Strasbourg, CNRS UMR, 7357, Strasbourg 67000, France; Department of Nuclear Medicine and Molecular Imaging, ICANS, Strasbourg 67000, France; Department of Neurosurgery, University Hospitals of Strasbourg, Strasbourg, 67091, France; MNMS Platform, University Hospitals of Strasbourg, Strasbourg 67098, France; ICube, University of Strasbourg, CNRS UMR, 7357, Strasbourg 67000, France; Department of Nuclear Medicine and Molecular Imaging, ICANS, Strasbourg 67000, France; Computer Engineering Department, Bilkent University, 06800 Ankara, Turkey; Computational Biology Department, Carnegie Mellon University, Pittsburgh, PA 15213, United States

## Abstract

**Motivation:**

Online assessment of tumor characteristics during surgery is important and has the potential to establish an intra-operative surgeon feedback mechanism. With the availability of such feedback, surgeons could decide to be more liberal or conservative regarding the resection of the tumor. While there are methods to perform metabolomics-based tumor pathology prediction, their model complexity predictive performance is limited by the small dataset sizes. Furthermore, the information conveyed by the feedback provided on the tumor tissue could be improved both in terms of content and accuracy.

**Results:**

In this study, we propose a metabolic pathway-informed deep learning model (PiDeeL) to perform survival analysis and pathology assessment based on metabolite concentrations. We show that incorporating pathway information into the model architecture substantially reduces parameter complexity and achieves better survival analysis and pathological classification performance. With these design decisions, we show that PiDeeL improves tumor pathology prediction performance of the state-of-the-art in terms of the Area Under the ROC Curve by 3.38% and the Area Under the Precision–Recall Curve by 4.06%. Similarly, with respect to the time-dependent concordance index (c-index), PiDeeL achieves better survival analysis performance (improvement of 4.3%) when compared to the state-of-the-art. Moreover, we show that importance analyses performed on input metabolite features as well as pathway-specific neurons of PiDeeL provide insights into tumor metabolism. We foresee that the use of this model in the surgery room will help surgeons adjust the surgery plan on the fly and will result in better prognosis estimates tailored to surgical procedures.

**Availability and implementation:**

The code is released at https://github.com/ciceklab/PiDeeL. The data used in this study are released at https://zenodo.org/record/7228791.

## 1 Introduction

Gliomas have uncertain patient mortality rates due to differences in suspected brain cell types and the variance observed in cancer progression dynamics (Omuro and DeAngelis 2013). 2021 version of central nervous system tumor taxonomy developed by the World Health Organization (WHO) (Osborn *et al.* 2022) categorizes gliomas according to cell types (e.g. astrocytes, oligodendrocytes) as well as four pathologically different grades (i.e. grade I–IV). This taxonomy takes differential histopathologic, molecular, and genetic characteristics of gliomas into account and orders grades with respect to increasing malignancy and decreasing prognostic optimism. Considering the prevalence of gliomas among brain tumors diagnosed in adults (Ostrom *et al.* 2017), devising a surgical management strategy that aims to make personalized clinical decision-making plausible via an intraoperative or preoperative surgeon feedback mechanism becomes essential.

In order to amplify the percentage of tumor tissue resected from a patient’s brain, various imaging (Stummer *et al.* 2011), spectroscopy as well as mass spectrometry (Schäfer *et al.* 2011, Brown *et al.* 2012, Eberlin *et al.* 2013, Santagata *et al.* 2014, Calligaris *et al.* 2015a,b, Fatou *et al.* 2016, Jarmusch *et al.* 2016, Pirro *et al.* 2017), and optical spectrometry (Stummer *et al.* 2006, Tsugu *et al.* 2011, Colditz and Jeffree 2012, Montcel *et al.* 2013, Li *et al.* 2014, Jermyn *et al.* 2016, 2017, Lu *et al.* 2016, Orringer *et al.* 2017, Poulon *et al.* 2017, Chan *et al.* 2018, Hollon *et al.* 2018, Xue *et al.* 2018) based techniques, were proposed in the literature. As a member of this cohort, ^1^H High-Resolution Magic Angle Spinning Nuclear Magnetic Resonance (HRMAS NMR) spectroscopy measures metabolic activity in a given unprocessed tissue specimen (Gogiashvili *et al.* 2019) with a turnaround time of ∼20 min and generates a time domain signal (Free Induction Decay–FID) in the form of the sum of decaying exponential functions. The FID signal is often preprocessed to obtain the HRMAS NMR spectrum defined on the frequency domain.

After preprocessing, the HRMAS NMR spectrum becomes well-suited to analyze the presence of left-over tumor tissues in the excision cavity and provide feedback to the surgeon about the nature of the tumor. Establishing this HRMAS NMR-guided intraoperative feedback loop requires a pathologist knowledgeable about tumor metabolism, and an NMR technician who detects an abundance of a few metabolites from the spectrum. However, the feedback resulting from this strategy is limited by (i) the availability of the experts during the surgery timeframe; and (ii) the throughput of the metabolite quantification procedure manually performed by the technician one metabolite at a time for HRMAS NMR spectra obtained from each tissue specimen. Moreover, overlapping metabolite peaks may not be fully separated in the preprocessed ^1^H spectra (Karakaslar *et al.* 2020).

Due to the high-dimensional, complex, and structured nature of the preprocessed HRMAS NMR spectra, data-driven computational modeling (i.e. machine learning) has the promise to be a valuable companion to the above-mentioned manual feedback loop. To this end, various supervised machine learning models were explored for providing automated feedback on pathological classification (Cakmakci *et al.*, 2020b, 2022) of the resected glioma tissue specimens. The dataset used for these studies (Cakmakci *et al.* 2020a) consists of HRMAS NMR spectra of 568 glioma and healthy control tissue samples. It is relatively small despite being the largest of its kind which prohibited the use of complex models and forced the models built to be shallow. This suggests room for improvement in the performance of the feedback pipeline.

From a methodological perspective, the applicability of deep learning models in small data regimes can be improved by making informed architectural choices to simplify models. Incorporating biological prior information may limit the connectivity of the neurons in a model and simultaneously (i) alleviate overfitting when available dataset sizes are small, and (ii) aid model interpretability regardless of the dataset size. While there is no application in the metabolomics domain, biologically informed deep learning architectures were successfully utilized in the context of metastasis prediction in patients with prostate cancer based on their genomic profiles (Elmarakeby *et al.* 2021), phenotype prediction based on the genetic variants of the patients (van Hilten *et al.* 2021), long-term survival prediction in patients with glioblastoma, based on multi-omics data (Oh *et al.* 2021), and multi-modal data integration (Liu *et al.* 2023).

Furthermore, the pathological classification of tumors may not be the only determinant of multi-faceted and patient-specific cancer progression semantics. In the case of glioblastoma multiforme (GBM), a grade IV astrocytic glioma type, a dismal prognosis is often expected due to the highly malignant and invasive nature of this class of tumors (Omuro and DeAngelis 2013). Despite their severity, prolonged and progression-free survival was observed in isocitrate dehydrogenase wildtype GBM (Burgenske *et al.* 2019, Wong and Yip 2019, Poon *et al.* 2020). This finding promotes the inclusion of survival risk estimates in the feedback given to surgeons for more accurate surgical decision-making. Recently proposed deep learning models for survival prediction include DeepSurv (Katzman *et al.* 2018), DeepHit (Lee *et al.* 2018), PC-Hazard (Kvamme and Borgan 2021), and Surv-Net (Wang *et al.* 2020).

**Figure 1.**
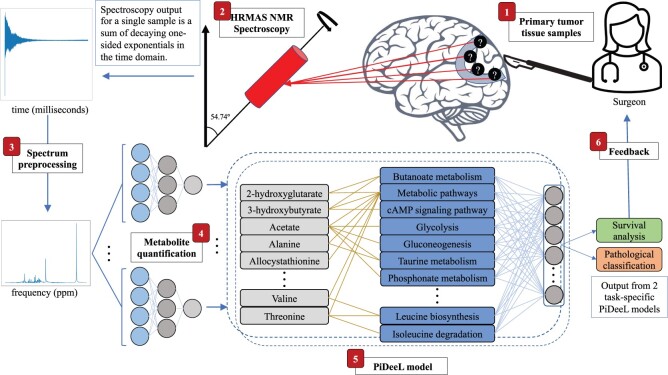
PiDeeL assisted intraoperative feedback loop. (1) The surgeon removes primary tumor tissue from the patient’s brain and prepares specimens from the resected tissue. (2) Prepared samples are sent for HRMAS NMR spectroscopy analysis. (3) Time domain signals resulting from the spectroscopy of each sample are preprocessed to uncover metabolite peaks harbored in the corresponding frequency domain spectrum. (4) Thirty-seven metabolites are quantified from preprocessed spectra in the order of milliseconds via metabolite-specific two-layer fully connected neural networks. (5) PiDeeL transforms metabolite concentrations to survival analysis and pathological classification predictions also in the order of milliseconds. (6) Based on this feedback, the surgeon decides to preempt or continue performing the surgery, again with the guidance of PiDeeL.

Here, we propose a deep neural network architecture (pathway-informed deep learning model, PiDeeL) to predict the survival of the glioma patient and the pathological classification of the tumor using the HRMAS NMR signal to give feedback to the surgeons (Fig. 1). While there were methods proposed for automated pathological classification, this is the first study to incorporate these two orthogonal sources of information to the surgeon as feedback at the same time. For the first time in the metabolomics domain, we incorporate prior biological information from the metabolic networks into the neural network architecture and increase the interpretability of the predictions which is also important for the surgeon to understand the underlying biology. We show that PiDeeL achieves better performance compared to the state-of-the-art model in pathological classification and, provides survival prediction with a c-index of 68.7%. We also analyze the most important metabolites and metabolic pathways for the reasoning of the model and discuss their relevance in the context of gliomas.

## 2 Materials and methods

### 2.1 Dataset

The dataset used in this study is a subset of the patient cohort released at https://zenodo.org/record/5781769, and it consists of 568 HRMAS NMR spectra acquired from 458 glioma (80.7% aggressive and 19.3% benign) and 110 control tissue specimens collected during respective surgeries of 513 patients performed at University Hospitals of Strasbourg. For the details of tissue specimen collection, and ethics statement, please see Supplementary Section S1.1 of the Supplementary Text.

In order to prepare the dataset for survival analysis, we retain the samples acquired from primary tumor tissue specimens of glioma origin whose record of the date of metabolomics-guided surgery and the date of decease or last control is present. We removed specimens resected from healthy control patients and excision cavities of glioma patients. The resulting set of 384 HRMAS NMR spectra contains one sample per glioma patient and is composed of 299 (77.9%) aggressive and 85 (22.1%) benign tumors whose subtype and grade information are provided in Supplementary Table S1. 70.1% of the patient cohort (269 patients) was reported deceased before the spectra acquisition from tissue specimens (i.e. until January 2021). The remaining 115 patients were considered to be censored as their survival status is not known. The distribution of patient age and time-to-event for deceased and censored patients are provided in panels A, B, and C of Supplementary Fig. S1, respectively. Furthermore, ERETIC-CPMG HRMAS NMR FID signals of these 384 glioma patients, whose spectra were acquired according to the procedure described in Supplementary Section S1.1.3 of Supplementary Text, are released at https://zenodo.org/record/7228791.

### 2.2 Problem formulation

Here, we formulate pathological classification and survival analysis tasks based on the full output of the metabolite quantification pipeline described in Supplementary Section S1.2 of the Supplementary Text as well as tumor malignancy label, patient-specific survival event indicator (i.e. censored or deceased), and time-to-event variables.

For a given sample *i*, we denote the feature vector, tumor malignancy label, survival event indicator, and time-to-event as ${\hat{M}}^{\left(i\right)}$, $Y_{m}^{\left(i\right)}$, $Y_{e}^{\left(i\right)}$, and $Y_{d}^{\left(i\right)}$, respectively. The feature vector, ${\hat{M}}^{\left(i\right)}\in R^{37}$, is the 37D vector corresponding to the full output of the automated metabolite quantification pipeline. For the complete list of 37 metabolites, details of automated metabolite quantification, and HRMAS NMR spectrum preprocessing, please see Supplementary Section S1.2 of Supplementary Text. Formally, let the predicted concentration of a given metabolite *j* for a sample set of size *N* be ${\hat{M_{j}}}^{\left(1:N\right)}\in R^{N\times 1}$. We denote the full output of the automated metabolite quantification pipeline for 37 metabolites and a sample set of size *N* as the concatenation of all predicted quantification vectors across metabolites:

(1) $$\begin{array}{cc} {\hat{M}}^{\left(1:N\right)} & =[{\hat{M}}_{1}^{\left(1:N\right)},{\hat{M}}_{2}^{\left(1:N\right)},\ldots ,{\hat{M}}_{37}^{\left(1:N\right)}]\in R^{N\times 37} \end{array}$$

Survival event indicator, $Y_{e}^{\left(i\right)}\in \{0,1\}$, is a binary variable that indicates whether the sample is from a deceased (i.e. 1) or a censored patient (i.e. 0). Time to survival event, $Y_{d}^{\left(i\right)}\in \left[0,\infty \right]$ is a nonnegative continuous variable defined as the time (in days) between metabolomics-guided tumor removal surgery and the date of decease or last control for deceased and censored patients, respectively. Finally, the tumor malignancy label, $Y_{m}^{\left(i\right)}\in \{0,1\}$, indicates whether the sample is from a tumor having aggressive (i.e. 1) or benign (i.e. 0) pathology, similar to the problem formulations presented in Cakmakci *et al.* (2020b, 2022).

For our formulation of the survival analysis task, we refer to the hazard function denoted as $\lambda (t,{\hat{M}}^{\left(i\right)})$ for a given sample *i* where *t* is time-to-event (i.e. $Y_{d}^{\left(i\right)}$). Assuming proportional hazards from Cox’s model (Cox 1972), the hazard function factorizes (Faraggi and Simon 1995, Katzman *et al.* 2018) to a time-dependent baseline hazard function, $h_{0}(t)$, and a sample-dependent risk function, $h({\hat{M}}^{\left(i\right)})$, as given in Equation 2.

(2) $$\lambda (t,{\hat{M}}^{\left(i\right)})=h_{0}(t)\cdot e^{h({\hat{M}}^{\left(i\right)})}$$

We train survival analysis models based on this formulation of hazard function on a dataset of *N* samples to learn a mapping function $f:R^{37}\to \left[-\infty ,\infty \right]$ such that $f({\hat{M}}^{\left(1:N\right)})=h({\hat{M}}^{\left(1:N\right)})$. Here, the goal of the trained models is to predict risk scores for glioma patients based on their metabolic profile. In order to infer these models, we make use of $Y_{e}^{\left(i\right)}$ and $Y_{d}^{\left(i\right)}$ as labels.

On the other hand, we train pathological classification models on a dataset of *N* samples to learn a mapping function $g:R^{37}\to \{0,1\}$ such that $g({\hat{M}}^{\left(1:N\right)})=Y_{m}^{\left(1:N\right)}$ [a binary classification problem similar to Cakmakci *et al.* (2020b, 2022)].

### 2.3 Baseline models

In this section, we provide the details of models benchmarked against PiDeeL for survival analysis and pathological classification tasks formulated in Section 2.2.

#### 2.3.1 Survival analysis

For the survival analysis task, we consider Cox Proportional Hazards (Cox-PH), Component-wise Gradient Boosting (CWGB), Random Survival Forest (RSF), DeepHit (Lee *et al.* 2018), PC-Hazard (Kvamme and Borgan 2021), and DeepSurv (Katzman *et al.* 2018) models as baselines.

Cox-PH model is a linear model that assumes proportional hazards to factorize instantaneous hazard function as a product of a baseline hazard function and a sample-dependent risk function, $h({\hat{M}}^{\left(i\right)})$, as formulated in Section 2.2. Based on the formulation provided by Cox (1972), the risk function estimate for sample *i*, ${\hat{h}}_{\beta }({\hat{M}}^{\left(i\right)})$, is provided by the linear model given in Equation 3.

(3) $${\hat{h}}_{\beta }({\hat{M}}^{\left(i\right)})=\beta^{t}{\hat{M}}^{\left(i\right)}$$

where ${\hat{M}}^{\left(i\right)}$ is the feature vector of metabolite concentrations for sample *i*, and β is the coefficient vector. The model is fit by maximizing Cox partial likelihood (Cox 1972, Faraggi and Simon 1995, Katzman *et al.* 2018) given in Equation 4.

(4) $$L(\beta )=\prod_{i:Y_{e}^{\left(i\right)}=1}\frac{\;\text{exp}({\hat{h}}_{\beta }({\hat{M}}^{\left(i\right)}))}{\sum_{j:Y_{d}^{\left(j\right)}>Y_{d}^{\left(i\right)}}exp({\hat{h}}_{\beta }({\hat{M}}^{\left(j\right)})))}$$

The gradient boosting approach iteratively performs rounds of boosting to improve the prediction performance of an ensemble of base learners (e.g. linear model). In each round, new base learners are trained to minimize the gradient of prediction error of the current ensemble model, and the best-performing candidate is added to the ensemble. In our context, we focused on the component-wise variant of gradient boosting, which adopts component-wise least squares as the base learner of choice (Hothorn *et al.* 2006). Under the proportional hazards assumption, the instantaneous hazard function factorizes as given in Equation 2.

RSF (Ishwaran *et al.* 2008) is a nonlinear ensemble method that constructs a predefined number of survival trees using a random subset of features and bootstraps of training data to estimate the cumulative hazard function, Λ(t), defined in Equation 5. Internal nodes of survival trees are split in order to maximize log-rank test (Segal 1988, LeBlanc and Crowley 1992) statistic between resulting child nodes.

(5) $$\Lambda (t)=\int_{0}^{t}\lambda (u)du$$

Neural network-based approaches to survival analysis are introduced by Faraggi and Simon (1995). Deeper variants of these models are more recently proposed as DeepSurv (Katzman *et al.* 2018), DeepHit (Lee *et al.* 2018), and PC-Hazard (Kvamme and Borgan 2021).

DeepHit is a multi-layer perceptron that considers time to be discrete and models competing risks. As we have only one event type, we consider DeepHit-Single (hereafter DeepHit) for our task. The model discretizes the event times to create a uniform grid of time intervals between the duration threshold in the sample set. The model assumes time is discrete from $T_{0}$ to $T_{\mathrm{final}}$. Let $\hat{h}(\hat{M})$ = ${\hat{h}}_{0}(\hat{M})$ + ⋯ + ${\hat{h}}_{\mathrm{final}}(\hat{M})$ show the estimated hazard (probability) mass function as output from the model. The survival function estimated by the model is shown in Equation 6.

(6) $$\hat{S}(T_{j}|\hat{M})=1-\sum_{k=1}^{j}{\hat{h}}_{k}(\hat{M})$$

The model has *m* output neurons with linear activation where *m* is a tunable hyperparameter and adopts two loss functions, namely, a discrete negative log-likelihood loss ($\mathrm{los}s_{L}$) and a ranking loss ($\mathrm{los}s_{\mathrm{rank}}$) which are shown in Equations (7) and (8), respectively.

(7) $$\mathrm{los}s_{L}=-\sum_{i=1}^{N}[Y_{e}^{\left(i\right)}\;\text{log}({\hat{h}}_{Y_{d}^{\left(i\right)}}({\hat{M}}^{\left(i\right)}))+(1-Y_{e}^{\left(i\right)})\;\text{log}(\hat{S}(T_{i}|{\hat{M}}^{\left(i\right)}))]$$

(8) $$\mathrm{los}s_{\mathrm{rank}}=\sum_{j:Y_{d}^{\left(j\right)}>Y_{d}^{\left(i\right)}}Y_{e}^{\left(i\right)}\;\text{exp}(\frac{\hat{S}(T_{i}|{\hat{M}}^{\left(i\right)})-\hat{S}(T_{i}|{\hat{M}}^{\left(j\right)})}{\sigma })$$

where σ is a tunable hyperparameter. The loss function of DeepHit is given in Equation (9), where α is used to combine the two losses.

(9) $$\mathrm{loss}=\alpha \mathrm{los}s_{L}+(1-\alpha )\mathrm{los}s_{\mathrm{rank}}$$

PC-Hazard (Kvamme and Borgan 2021) is a multi-layer perceptron that parametrizes the continuous-time likelihood contribution function. The model considers hazard as a piecewise constant. Let time is partitioned from $T_{0}$ to $T_{\mathrm{final}}$. Then, the hazard function h(t) is a step function for some nonnegative constants $\left(\eta_{1},\ldots ,\eta_{\mathrm{final}}\right)$ as given in Equation (10).

(10) $$h(t)=\eta_{\kappa (t)}$$

ρ(t) is the interval fraction of κ(t) at time *t* and formulated as given in Equation (11).

(11) $$\rho (t)=\frac{t-T_{\kappa (t)-1}}{\Delta_{T_{\kappa (t)}}}$$

The model has *m* output neurons with linear activation and adopts a mean negative log-likelihood loss as given in Equation (12), where $\hat{\eta }$ is the estimated vector of constants.

(12) $$\mathrm{loss}=-\frac{1}{N}\sum_{i=1}^{N}\left(Y_{e}^{\left(i\right)}\;\text{log}\;{\hat{\eta }}_{\kappa (t_{i})}{\hat{M}}^{\left(i\right)}-{\hat{\eta }}_{\kappa (t_{i})}{\hat{M}}^{\left(i\right)}\rho (t)-\sum_{j=1}^{\kappa (t_{i})-1}{\hat{\eta }}_{j}{\hat{M}}^{\left(i\right)}\right)$$

DeepSurv is a multi-layer perceptron that models sample-specific risk function, $h({\hat{M}}^{\left(i\right)})$, as formulated in Section 2.2. The model has a single output neuron with linear activation and adopts a negative log of Cox partial likelihood given in Equation (13) as the loss function.

(13) $$\text{log}(L(\beta ))=-\sum_{i:Y_{e}^{\left(i\right)}=1}\left({\hat{h}}_{\beta }({\hat{M}}^{\left(i\right)})-\text{log}\;\sum_{j:Y_{d}^{\left(j\right)}>Y_{d}^{\left(i\right)}}e^{\left({\hat{h}}_{\beta }({\hat{M}}^{\left(j\right)})\right)}\right)$$

We consider two-, three-, and four-layer DeepHit, PC-Hazard, and DeepSurv models as baseline models. We use a single neuron (multiple for DeepHit, and PC-Hazard) in the last layer with linear activation to output the predicted risk scores of samples. The trained three-layer DeepHit, PC-Hazard, and DeepSurv models on our dataset of size *N* can be summarized as follows:

(14) $$\begin{array}{cc} X_{\mathrm{hid}}^{\left(1:N\right)} & =\mathrm{ReLU}(FC^{\left(64\right)}({\hat{M}}^{\left(1:N\right)})) \end{array}$$

(15) $$X_{\mathrm{hid}}^{\left(1:N\right)}=\mathrm{ReLU}(FC^{\left(64\right)}(X_{\mathrm{hid}}^{\left(1:N\right)}))$$

(16) $$\begin{array}{cc} {\hat{h}}^{\left(1:N\right)} & =FC^{\left(1\right)}(X_{\mathrm{hid}}^{\left(1:N\right)}) \end{array}$$

where $X_{\mathrm{hid}}$ represents the output of the first hidden layer, ${\hat{h}}^{\left(i\right)}$ represents the predicted risk score, $FC^{\left(\cdot \right)}$ represents a fully connected layer with (⋅) neurons, and *ReLU* represents the rectified linear unit. In the case of two- and four-layer DeepHit, PC-Hazard, and DeepSurv models, we remove or repeat the hidden layer defined in Equation (15). For details of the baseline neural networks, please see Supplementary Section S1.4.7 of the Supplementary Text. We also consider the DeepSurv model that takes the full spectrum as input. Please see Supplementary Section S1.4.5 of the Supplementary Text for the details of this baseline model and Supplementary Section S1.4.1 for the baseline models for the pathological classification task.

### 2.4 Pathway-informed deep learning model

In this section, we introduce PiDeeL, a pathway-informed multi-layer perceptron model designed to integrate biomarker metabolite concentrations provided as input at the metabolic pathway level. We use the Kyoto Encyclopedia of Genes and Genomes (KEGG) database to parse the metabolite-to-pathway mapping and construct a 37×138 matrix, hereafter PI_matrix, where the value in the *i*th row and *j*th column of matrix PI_matrix is 1 if the metabolite *i* appears in the pathway *j*, and 0 otherwise. Similar to the baseline fully connected neural network models for the survival analysis and pathological classification tasks, we consider two-, three-, and four-layer PiDeeL models.

#### 2.4.1 Model architecture

Given a dataset of *N* samples, the architecture of the three-layer PiDeeL model for the survival analysis can be summarized as follows:

(17) $$\begin{array}{cc} PL^{\left(138\right)} & =\left[\begin{array}{c} FC^{\left(138\right)}w\mathrm{eights} \end{array}\right]\left[\begin{array}{c} PI\_\mathrm{matrix} \end{array}\right] \end{array}$$

(18) $$PA^{\left(1:N\right)}=\mathrm{ReLU}(PL^{\left(138\right)}({\hat{M}}^{\left(1:N\right)})+b^{138})$$

(19) $$\begin{array}{cc} X_{\mathrm{hid}}^{\left(1:N\right)} & =\mathrm{ReLU}(FC^{\left(64\right)}(PA^{\left(1:N\right)})) \end{array}$$

(20) $${\hat{h}}^{\left(1:N\right)}=FC^{\left(1\right)}(X_{\mathrm{hid}}^{\left(1:N\right)})$$

where $PL^{\left(138\right)}$ and $b^{138}$ represent the weights and biases of the pathway-informed layer, $PA^{\left(1:N\right)}$ represents the activations of the first hidden layer (hereafter pathway-activation), $X_{\mathrm{hid}}$ represents the output of hidden layers, ${\hat{h}}^{\left(i\right)}$ represents the predicted risk score, $FC^{\left(\cdot \right)}$ represents a fully connected layer with (⋅) neurons and *ReLU* represents the rectified linear unit. We incorporate the metabolic pathway information into PiDeeL by defining the first layer of the model using PI_matrix. This architectural decision makes the first layer of PiDeeL sparse since the connections in the first layer are only permitted to be formed between input metabolites and the pathways they are involved in. We use the negative log of Cox Partial Likelihood as the loss function, similar to the DeepSurv models formulated in Section 2.3.1, in order to train PiDeeL for the survival analysis task. We also train PiDeeL with DeepHit and PC-Hazard losses. We find that the best-performing loss function for PiDeeL is the DeepSurv loss. Please see Supplementary Section S1.4.6 of Supplementary Text and Supplementary Fig. S8 for these experiments.

The architecture of PiDeeL used for survival analysis and pathological classification tasks differs only in the output layer [i.e. Equation (20)]. In order to adapt PiDeeL for pathological classification we use the sigmoid function as the output activation function by replacing Equation (20) with Equation (21). In addition, we use class-weighted binary cross entropy as the loss function.

(21) $$\begin{array}{cc} {\hat{Y_{m}}}^{\left(1:N\right)} & =\mathrm{Sigmoid}(FC^{\left(1\right)}(X_{\mathrm{hid}}^{\left(1:N\right)})) \end{array}$$

#### 2.4.2 Interpretability and importance analyses

We perform SHapley Additive exPlanations (SHAP) analysis on our feature vector (${\hat{M}}^{\left(1:N\right)}$), and pathway activations ($PA^{\left(1:N\right)}$) to assess the importance of each metabolite and pathway on the prediction of PiDeeL. For the details of these analyses, please see Supplementary Section S1.4.3 of the Supplementary Text.

#### 2.4.3 Ablation studies for pathway-informed architecture

In order to verify that the improvements achieved by PiDeeL over baseline models are due to the pathway-informed architecture, we conduct a series of ablation tests as follows:

- We train fully connected models with 138 neurons in the first hidden layer to check whether the number of neurons in the first layer (138) of PiDeeL contributes to the performance.
- We train PiDeeL models using randomly connected PI_matrix instead of PI_matrix that are constructed from the KEGG database to check whether the pathway-informed connections have a benefit over the same number of randomly connected edges.
- We train PiDeeL models using shuffled PI_matrix. This is similar to randomly connected PI_matrix except it preserves the in-degrees of pathways.
- We train the DeepSurv model with different dropout rates (0.5,0.6,0.7,0.8,0.9) in the first hidden layer to check whether the pathway-informed layer has a benefit over this widely used technique to regularize neural networks.
- We train and test DeepSurv and PiDeeL on sample sets of sizes 50, 100, and 200 to test the models’ adaptabilities to different dataset sizes.

Please refer to Supplementary Section S1.4.4 of the Supplementary Text for the details of ablation studies for pathway-informed architecture based on survival analysis.

## 3 Results

### 3.1 Experimental setup

In this section, we describe the experimental setup used for evaluating PiDeeL as well as baseline models formulated in Section 2.3 in the context of the survival analysis scenario. Please see Supplementary Section S1.4.1 of the Supplementary Text for the results of the pathological classification. We measure the performance of survival analysis models in the unit of time-dependent concordance index (c-index) described in Antolini *et al.* (2005). The original concordance index (Harrell *et al.* 1982) is computed with the individually predicted patient risk scores, whereas, with a time-dependent definition, individual risk scores are calculated with the predicted risk function and the baseline hazard function, i.e. computed on the sample set (Antolini *et al.* 2005, Gerds *et al.* 2013).

For each task and model type, we adopt a 5-fold cross-validation routine and repeat it three times with different seeds (i.e. 15 iterations). Before each iteration, we shuffle and divide our dataset into training, validation, and test set such that no sample or patient is shared across folds. The results discussed hereafter are with respect to the predictive capability of models on the test split. During iteration *i* of the cross-validation setup, Cox-PH, CWGB, and RSF models were tested on the fold *i*, validated on fold (i+1)mod5 and trained on the remaining three folds. We re-train the selected configuration on the union of training and validation splits to get stronger baseline models. For all deep learning models, re-training steps are not performed due to the presence of early-stopping dynamics. Please see Supplementary Section S1.3 of Supplementary Text for hyperparameter space and selection details.

### 3.2 Survival analysis

**Figure 2.**
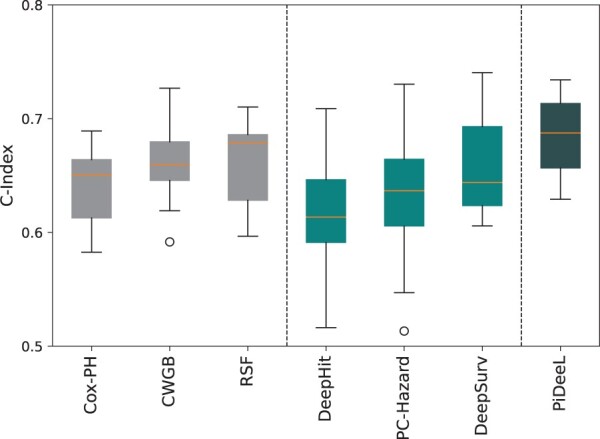
Comparison of baseline models and PiDeeL on survival prediction in terms of c-index. This evaluation is based on a 5-fold cross-validation, i.e. repeated three times. (i.e. 15 iterations).

Please see Fig. 2 for the survival prediction performances of (i) Cox-PH, CWGB, and RSF models (gray boxplots); (ii) DeepHit, PC-Hazard, and DeepSurv models (cyan boxplots); and PiDeeL model (navy blue boxplot), with respect to the c-index. Note that in Fig. 2 the performance of four-layer neural network models (DeepHit, PC-Hazard, DeepSurv, and PiDeeL) are shown. Please see Supplementary Section S1.4.7 of the Supplementary Text and Supplementary Figs S9–S11 for the performance comparison of two-, three-, and four-layered DeepHit, PC-Hazard, and DeepSurv models against PiDeeL. We observe that PiDeeL outperforms other methods and achieves a median c-index of 68.7%. RSF is a particularly strong baseline with a median c-index of 67.8%. Among the other neural network-based models, DeepSurv achieves the highest median c-index 64.3%.

To show the advantage of using the automated metabolite quantification pipeline, we also compare PiDeeL with a version of the DeepSurv that inputs the full HRMAS NMR spectrum instead of quantified metabolites (see Supplementary Section S1.4.5 of the Supplementary Text). PiDeel outperforms this baseline in terms of median c-index by 7.9%, 8.3%, and 5.0% for two-, three-, and four-layered models, respectively. Note that it is not possible to use PiDeel directly with the full NMR spectrum, as incorporating the pathway information to the model is not straightforward in that case.

### 3.3 Interpretability and importance analyses

When combined with downstream feature importance analysis methods, [SHAP (Lundberg and Lee 2017)], the pathway-informed architecture of PiDeeL enables us to jointly analyze the importance of metabolites and pathways for the model output. In addition to the quantitative analyses presented above, we perform feature importance analyses on the four-layer PiDeeL to empirically pinpoint metabolites and pathways candidate for being implicated in gliomas, their progression, and associated patient survival. We calculate SHAP values on the input features (i.e. *in silico* predicted metabolite concentrations—${\hat{M}}^{\left(1:N\right)}$) as well as pathway-informed layer activations ($PA^{\left(1:N\right)}$).

**Figure 3.**
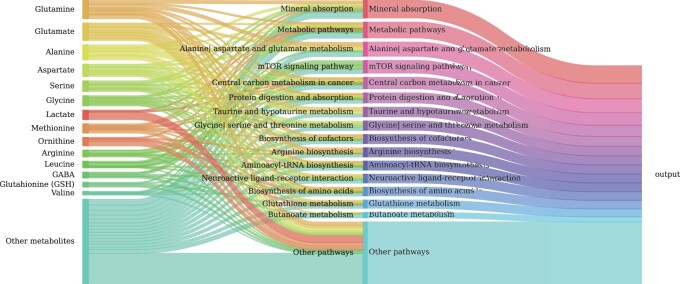
Sankey diagram to visualize the relative importance of metabolites and pathways for PiDeeL. Nodes on the left represent metabolite level inputs, nodes in the middle represent pathway layer activations, and the node on the right represents the output of the model. Larger nodes are relatively more important for the model, while thinner nodes are less important. A flow in the diagram depicts the contribution of a metabolite to a pathway. A larger flow represents a bigger contribution to the next layer node. For example, the contribution of Glutamine to Mineral absorption pathway importance is larger than the contribution of Methionine to Protein digestion and absorption pathway importance.

Please see Supplementary Fig. S4 where we show PiDeeL’s metabolite importance plots for the survival prediction and tumor pathological classification tasks. We highlight the top three metabolites having the highest mean SHAP value for the survival analysis among all 37 metabolites according to our analyses in the decreasing order of importance *glutamate*, *glutamine*, and *alanine*. We also pinpoint the top three pathways having the highest SHAP values among the 138 pathways used for pathway-informed layer, in the decreasing order of importance as: *Mineral absorption*, *Alanine, aspartate and glutamate metabolism*, and *mTOR signaling pathway*. Please see Fig. 3 where we show the relative importance of metabolites and pathways on the outcome of PiDeeL. In this figure, we show the metabolites (on the left), pathways (in the middle), and the output (on the right) as nodes. The sizes of the nodes represent the importance of that particular metabolite or pathway to PiDeeL. The thickness of the flow from one metabolite to a pathway represents the contribution of that metabolite to the importance of the pathway. Please also see Supplementary Section S1.4.3 of the Supplementary Text for the biological motivation for interpretability and importance analyses.

### 3.4 Ablation studies for pathway-informed architecture

**Figure 4.**
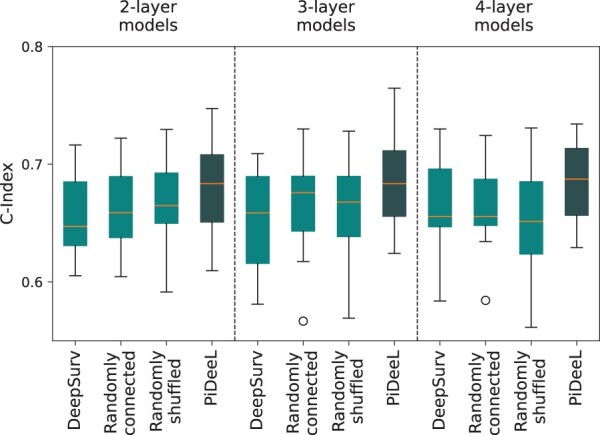
Performance comparison of PiDeeL on survival analysis task against DeepSurv with 138 neurons in the first hidden layer, PiDeeL with randomly connected PI_matrix, and PiDeeL with shuffled PI_matrix, with respect to c-index.

In this section, we present the results of ablation studies discussed in Section 2.4.3. Figure 4 demonstrates the result of the respective itemized ablation tests (excluding dropout).

We divide Fig. 4 into three panels to show the results of two-, three-, and four-layer models. Under each panel, the boxplots from left to right demonstrate the performance attained by three ablation tests (cyan boxplots) that are DeepSurv model with 138 neurons in the first layer, PiDeeL with randomly connected PI_matrix, PiDeeL with randomly shuffled PI_matrix and also PiDeeL (navy blue boxplots).

We observe that PiDeeL improves the median c-index over DeepSurv with 138 neurons in the first layer by 3.6%, 2.6%, and 3.6% for two-, three-, and four-layered networks, respectively. We also see that using PiDeeL over DeepSurv decreases the variance by an average of 29.7%. Note that the original DeepSurv model has 64 neurons in the first layer. This shows that not the larger number of neurons in the first layer but the pathway information leads to the performance improvement over DeepSurv.

We compare the performance of PiDeeL with PI_matrix against PiDeeL with random PI_matrix. In this setup, we randomly connect our metabolite nodes (input vectors) and pathway activation layer neurons. For this, we constructed randomly connected 37×138 matrices preserving the total number of connections in the original PI_matrix (468). We observe that PiDeeL improves the median c-index over PiDeeL with random PI_matrix by 3.7%, 1.2%, and 4.9% for two-, three-, and four-layered networks, respectively.

We show the performance comparison of PiDeeL with PI_matrix against PiDeeL with shuffled PI_matrix. This time, instead of randomly connecting the PI_matrix, we shuffle the rows to preserve each metabolite-to-pathway profile. We observe a similar trend as in Panel C. This time, PI_matrix improves the performance attained by using shuffled PI_matrix in terms of median c-index by 2.8%, 2.4%, and 4.9%. In addition, using PI_matrix decreases the variance by an average of 4.6%.

**Figure 5.**
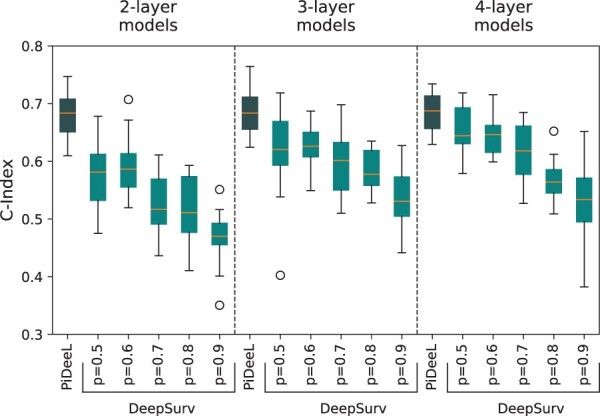
Performance comparison of PiDeeL on survival analysis task against DeepSurv with different dropout rates with respect to c-index. This evaluation is based on a 5-fold cross-validation, i.e. repeated three times. (i.e. 15 iterations).

Figure 5 shows the survival prediction performance comparison of DeepSurv with various dropout rates (d) and PiDeeL. We observe that PiDeeL substantially outperforms DeepSurv-with-dropout regardless of the rate. We also see that DeepSurv’s performance deteriorates when the dropout rate is increased. The two-layer PiDeeL model achieves a median c-index of 68.3%, in contrast, the DeepSurv model attains median c-index scores of 58.1%, 58.6%, 51.6%, 51.1%, and 47.0% for 0.5, 0.6, 0.7, 0.8, and 0.9 dropout rates, respectively. We observe the same trend as we increase the number of layers for both models. This indicates that sparsity provided by the dropout does not contribute to the performance of the fully connected model for survival prediction, whereas PiDeeL’s pathway-informed sparsity with PI_matrix improves the performance.

In Supplementary Fig. S7, we show the performance benchmark of PiDeeL and DeepSurv on different sample set sizes. We observe that on the sample set of size 200, PiDeeL improves the median c-index over DeepSurv by 5.4%, 7.6%, and 5.7% for two-, three-, and four-layered networks, respectively. For the sample set of size 100, PiDeeL outperforms DeepSurv with respect to the median c-index by 7.6%, 5.8%, and 11.8%. Finally, when we decrease the sample set size to 50, the performance gap between PiDeeL and DeepSurv increases substantially. PiDeeL improves the median c-index over DeepSurv by 4.9%, 13.5%, and 18.6% on the sample set of size 50. This shows that pathway-informed architecture improves performance and overcomes overfitting by decreasing the model complexity.

### 3.5 Validation on an independent dataset

**Figure 6.**
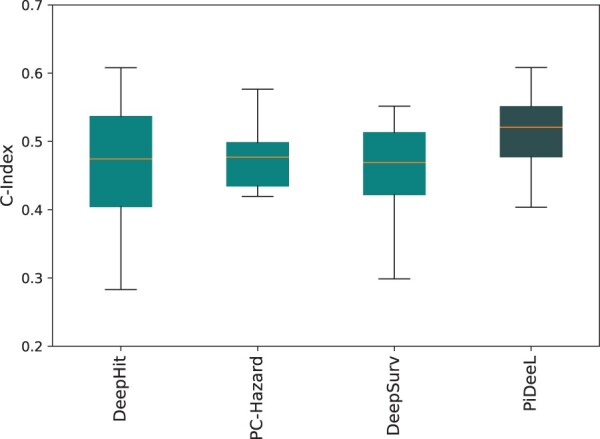
Performance comparison of PiDeeL and other models on an independent dataset with respect to c-index. This evaluation is based on 100 iterations with three different random seeds. (i.e. 300 total iterations).

We obtain an independent HRMAS NMR glioma dataset from Firdous *et al.* (2021). This dataset does not include survival information for the samples (i.e. event indicator $Y_{e}$ and/or time-to-event $Y_{d}$). The labels in the dataset consist of tumor grade (i.e. I–IV) and tumor subtypes (e.g. AST, GBM). We simulate $Y_{e}^{\left(i\right)}$ and $Y_{d}^{\left(i\right)}$ for sample (i) in the dataset of 26 tumor samples considering their grades and subtypes. Please see Supplementary Section S1.4.8 of the Supplementary Text for details of data simulation. We predict the metabolite quantities for 26 tumor samples in the dataset using their FID signals. Then, we test other neural network models and PiDeeL on this simulated data. Please see Fig. 6 for the performance benchmark of baseline models and PiDeeL. Median c-indices of baseline other models are under 50%.PiDeeL outperforms others with a median c-index of 52.1% and decreases the variance by an average of 15.2%.

## 4 Discussion

^1^H HRMAS NMR spectrum has been a reliable resource for distinguishing malign and healthy samples taken from the excision cavity during surgery. It is a good fit because it is nondestructive and can analyze small samples of raw tissue specimens (Gogiashvili *et al.* 2019). Machine learning techniques that learn from the NMR signal to distinguish healthy and tumor tissue, as well as benign and aggressive tumors, have been an important tool. Yet, the performance was limited due to small training set sizes (Cakmakci *et al.* 2020b, 2022) which prohibit using complex architectures like deep neural networks learning a hierarchical composition of complex features. In this study, we are able to use such a hierarchically complex model for distinguishing benign and aggressive tumors for the first time with comparable performance. Despite having a deep model, we are able to decrease the number of trainable parameters in the model by composing the architecture with prior biological information obtained from metabolic networks.

The pathology of the tumor is an important cue for the surgeon who might want to resect more tumor tissue and risk the patient’s well-being if the tumor is likely aggressive, or who might risk leaving some residual tumor tissue if the tumor is likely benign. Yet, this does not one-to-one determine the prognosis. Thus, it is also important to estimate the survival of a patient, which is a proxy for prognosis, and inform the surgeon. For this, we train a pathway-informed model to predict the survival of patients and show that we attain high performance. An example showing the usefulness of the survival prediction along with pathological classification is as follows: Our model is able to correctly predict long survival for a patient with aggressive glioma, who lived 5656 days after surgery. The surgeon can be more conservative in this case compared to being informed with only a prediction of an aggressive tumor. Similarly, our model correctly predicts low survival for a patient with a benign tumor who lived only 43 days after surgery.

We also investigate the use of a multi-task learning architecture that combines two architectures and predicts both labels simultaneously. Our results demonstrate that this approach only slightly increases the performance for both tasks. For this reason, we ultimately decided to proceed with the utilization of two task-specific single-task PiDeeL models.

One limitation of our study is that it is bottlenecked by the automated metabolite quantification model which has been shown to have promising performance and generalizability. Yet, this component consists of individual multi-layered perceptrons for 37 metabolites for which a training dataset exists. We think our model would benefit from a more comprehensive spectrum of metabolites to predict the survival of a patient which is a complex outcome to approximate. The human metabolome roughly consists of 3000 metabolites and we are currently limited to using only 1% of this information source.

## Supplementary Material

btad684_Supplementary_Data

## Data Availability

The data underlying this article are available in Zenodo at https://dx.doi.org/10.5281/zenodo.7228791.
