# Supplementary material for: PiDeeL: metabolic pathway-informed deep learning model for survival analysis and pathological classification of gliomas
Source: Bioinformatics. 2023 Nov 11;39(11):btad684. doi: 10.1093/bioinformatics/btad684 (PMC10663986; doi:10.1093/bioinformatics/btad684)
Supplement: btad684_Supplementary_Data [file btad684_supplementary_data.pdf]

Supplementary Material for PiDeeL: Metabolic Pathway-Informed deep learning model for survival analysis and pathological classification of gliomas

1 Supplementary Text

1.1 Dataset

1.1.1 Tissue Sample Collection

All tissue specimens were collected either by a pneumatic system connecting the operating theater of neurosurgery to the NMR room (Hautepierre hospital, University Hospitals of Strasbourg) or from samples stored at Strasbourg and Colmar tumor biobanks, with minimum ischemic delays (average time $2\,\text{min} \pm 1\,\text{min}$). The collected samples were adequate for proper HRMAS NMR analysis and had a low necrosis-to-tumor ratio. After HRMAS NMR analysis, inserts for half the content of each sample were cut. The percentage of tumor cells in the sample was calculated with respect to the total surface area as well as frozen hematoxylin & eosin-stained sections. Please see Supplementary Table 1 for the histopathological classification of the tumor tissue samples.

1.1.2 Ethics Statement

The tissue specimens used in this study were collected from samples stored in Strasbourg and Colmar tumor biobanks, or by a pneumatic system. Collection, storage, and compilation of the dataset were approved by the Ethics Committee no. 2003-100., Ethics Committee no. 2013-37, 12.11.2013. and Ethics Committee of Strasbourg, (Comité de protection des personnes, Est IV), respectively. Consent for all patients who participated in this study was acquired by a written letter.

1.1.3 HRMAS NMR Spectrum Acquisition

A Bruker Avance III 500 spectrometer equipped with a 4-mm triple-resonance gradient HRMAS probe (1H, 13C, and 31P) operating at a proton frequency of 500.13 MHz was used to acquire the spectra from biopsy samples prepared by combining $30\mu L$ KelF insert and 15- to 18-mg collected sample at 20°C. The lock frequency of the spectrometer was set by adding $10\mu L$ of D_2O to the insert. Spectrum acquisition was performed using a Carr-Purcell-Meiboom-Gill (CPMG) pulse sequence with a $285\mu s$ inter-pulse delay and 10 min acquisition time. The CPMG pulse train had a length of 93 ms due to 328 loops. During the spectrum acquisition process, the temperature was stabilized to 4°C in order to reduce the effects of tissue degradation. As a result, a one-dimensional (1D) proton (1H) spectrum was acquired for each specimen. Finally, a simulated,

Lorentzian-shaped ERETIC [2] signal, whose amplitude was calibrated to correspond to exactly 69.6 nmoles of protons, was added to the spectrum digitally at 10 ppm. The amplitude of the digitally added ERETIC signal also takes into account the difference in pulse widths between the standard acetate sample and the biopsy sample under investigation, making the method insensitive to the salt effect [21]. The calibrated ERETIC signal allowed the quantification of all the identified metabolites present in the spectrum.

1.2 Automated Metabolite Quantification

The automated metabolite quantification pipeline method presented in [6] was used to quantify the following 37 metabolites of interest: 2-hydroxyglutarate, 3-hydroxybutyrate, Acetate, Alanine, Allo-cystathionine, Arginine, Ascorbate, Aspartate, Betaine, Choline, Creatine, Ethanolamine, GABA, Glutamate, Glutamine, Glutathione (GSH), Glycerophosphocholine, Glycine, hypo-Taurine, Isoleucine, Lactate, Leucine, Lysine, Methionine, Myo-inositol, NAL, NAA, o-Acetylcholine, Ornithine, Phosphocholine, Phosphocreatine, Proline, scyllo-Inositol, Serine, Taurine, Threonine and Valine.

In order to prepare our HRMAS NMR spectra for this pipeline, we first remove the 70 initial time points in the FID signal which correspond to the spectroscopy machine-specific (Bruker for our case) digital filter. The clipped time domain signal was transformed to the frequency domain via Discrete Fourier Transform without windowing and phase-corrected after a line broadening of 10Hz was applied. The magnitude of the resulting signal (ranging from -2ppm to 12ppm) was normalized by tissue sample mass and multiplied with the constant acetate reference-based scaling factor provided in their paper. Finally, the scaled magnitude signal was binned to $1,401$ bins of 0.01 ppm-width by summing the intensity of the frequencies mapped to each bin. The resulting signal was used as input to the publicly available trained 2-layer fully-connected neural network-based quantification models available for each metabolite of interest from `https://github.com/gunkaynar/targeted_brain_tumor_margin_assessment`.

1.3 Experimental Setup

1.3.1 Hyperparameter Space

In this section, we give the lists of hyperparameter space for baseline models for both survival analysis and pathological classification tasks. In the of CoxPH model, we use a ridge-regularized variant to prevent overfitting. We perform hyperparameter optimization on the validation split. The hyperparameter search space was limited to: (1) regularization penalty coefficient $\in \{0, 0.1, ..., 0.9, 1\}$; (2) the heuristic for breaking ties in event ties was set to *efron* [8] or *breslow* [4]; and (2) the maximum number of iterations $\in \{100, 200, ..., 500\}$.

For the CWGB model, we perform hyperparameter selection on validation split using the following search space: the number of boosting stages to perform. $\in \{20, 50, 100, 150]\}$; loss function to be optimized. $\in \{"Cox - PH", "Squared"\}$; the percentage of base learners to be dropped $\in \{0.0, 0.1, 0.2\}$; learning rate $\in \{0.2, 0.1, 0.05\}$.

For the RSF model, similar to the case with Cox-PH and CWGB, we perform hyperparameter selection on validation split using the following search space: the number of trees in the forest $\in \{20, 50, 100, 150, 300, 400]\}$; the maximum depth limit for each tree $\in \{no\ limit, 10, 15, 25, 30\}$; the minimum number of samples required to split an internal node in a tree $\in \{2, 5, 10, 15\}$; the minimum number of samples required to be available in leaf nodes resulting from considered split

$\in \{2, 5, 10, 15\}$; and the maximum number of features explored while looking for best splitting criterion $\in \{sqrt(number\ of\ metabolites = 37), log_2(37)\}$.

DeepSurv model's hyperparameter search space is limited to: (1) batch size for training $\in \{16, 32, 64, 128, full-batch\}$; and (2) activation function between layers $\in \{ReLU, Sigmoid\}$.

On the other hand, for the pathological classification task, we have RF as the baseline model. In the case of RF models, we apply grid search for the hyperparameter selection and selected the setting with the best-observed performance (AUC-ROC) on the validation set. Hyperparameter search space for the RF model is as follows: the number of trees in the forest $\in \{50, 150, 300, 400]\}$; the maximum depth limit for each tree $\in \{10, 15, 25, 30\}$; the minimum number of samples required to split an internal node in a tree $\in \{5, 10, 15\}$; the minimum number of samples required to be available in leaf nodes resulting from considered split $\in \{2, 10, 20\}$; criterion to measure the quality of a split: *Gini-index* and *entropy*.

The other baseline model for the pathological classification task is fully-connected neural networks. The hyperparameter search space is similar to DeepSurv as follows: (1) batch size for training $\in \{16, 32, 64, 128, full-batch\}$; and (2) activation function between layers $\in \{ReLU, Sigmoid\}$.

1.3.2 Hyperparameter Selection

Regardless of the task, we use grid search on validation split to determine the best-performing hyperparameter values per model and iteration. Please see section 1.3.1 of Supplementary Text for the hyperparameter space in the case of baseline models. Training these models involved a two-step process. During iteration i of the cross-validation setup, these models were tested on the fold i, validated on fold $(i + 1) mod 5$, and trained on the remaining 3 folds. After the selection of the best-performing hyperparameter setting based on the c-index calculated on the validation set, we re-trained the selected configuration on the union of training and validation splits to get stronger baseline models.

Hyperparameters of deep learning-based models explored (DeepHit, PC-Hazard, and DeepSurv) or proposed (PiDeeL) for survival analysis were tuned based on the loss calculated on validation split predictions. Given its formulation in Section 2.3.1 of the manuscript, losses (Eq 9, 12, and 13 of the manuscript), we adopted for deep learning-based survival analysis are differentiable surrogates for time-dependent c-index metric [3]. Similar to the case with the survival models, hyperparameters of deep learning models for the pathological classification task were also tuned based on binary cross-entropy loss (which is also a differentiable proxy for AUC-ROC and AUC-PR) calculated on validation split. For all deep learning models discussed above, cross-validation folds were distributed to training, validation, and test splits similar to CoxPH, CWGB, and RSF models. However, re-training with the union of training and test splits step was not performed due to the presence of early-stopping dynamics. To form the hyperparameter space, we sampled 5 equidistant learning rates from $[10^{-3}, 10^{-4}]$ interval, performed experiments, and proceeded with 10^{-4} as the learning rate due to the highest empirically observed performance.

The hyperparameter search space for PiDeeL for both tasks was as follows: (1) batch size for training $\in \{16, 32, 64, 128, full-batch\}$; and (2) activation function between layers $\in \{ReLU, Sigmoid\}$. After conducting thorough experimentation with these hyperparameters, we determined the optimal settings for our model. Specifically, we selected batch sizes of 64 and 32 for survival analysis and pathological classification tasks, respectively. Additionally, we found that the use of the ReLU activation function resulted in the highest empirical performance, and thus, we proceeded with this choice for the activation function.

Moreover, we trained models at most $5,000$ epochs (selected from $1000, 2000, ..., 6000$) and applied early-stopping when validation loss increased for 150 consecutive epochs (selected among 50, 100, 150, 200). We used Adam optimizer [13] with a weight decay of 0.002, also selected based on the highest empirically observed performance.

The time discretization parameter *num_durations* for DeepHit and m for PC-Hazard are empirically selected as 10 among $\{5, 10, 20, 50, 100\}$.

1.3.3 Implementation Details

The codebase for this work is implemented in Python and is publicly available at `https://github.com/ciceklab/PiDeeL`. Specifically, RSF, CWGB, and CoxPH models were used from [17], and deep learning models were developed with the help of PyTorch framework [16]. All models are trained and tested on a SuperMicro SuperServer 4029GP-TRT with 2 Intel Xeon Gold 6140 Processors (2.3GHz, 24.75M cache), 251GB RAM, 6 NVIDIA GeForce GTX 2080 Ti GPUs (11GB, 352Bit) and 2 NVIDIA TITAN RTX GPUs (24GB, 384Bit). Training of CoxPh, RSF, and DeepSurv models took 1.5, 14, and 2 minutes on average. The training of PiDeeL took 1.5 minutes on average.

1.4 PiDeeL

1.4.1 Pathological Classification

We consider random forest (RF) from [6] and fully-connected neural networks as baseline models for the pathological classification task.

We consider neural network-based models for the pathological classification task as baseline models. We train 2, 3, and 4-layer fully-connected neural networks with class-weighted binary cross entropy loss. The trained 3-layer fully-connected networks on our dataset of size N can be summarized as follows:

$$X_{hid}^{(1:N)} = ReLU(FC^{(64)}(\widehat{M}^{(1:N)})) \tag{1}$$

$$X_{hid}^{(1:N)} = ReLU(FC^{(64)}(X_{hid}^{(1:N)})) \tag{2}$$

$$\widehat{Y_m}^{(1:N)} = Sigmoid(FC^{(1)}(X_{hid}^{(1:N)})) \tag{3}$$

where X_{hid} represents the output of the first hidden layer, $\widehat{Y_m}^{(1:N)}$ represents the predicted tumor pathology, $FC^{(\cdot)}$ represents a fully-connected layer with $(\cdot)$ neurons, and $ReLU$ represents the rectified linear unit. We also consider 2 and 4-layer fully-connected networks where we remove or repeat the hidden layer defined in Eq 2, respectively.

We assess the performance of the pathological classification models with respect to the Area Under the ROC Curve (AUC-ROC) and the Area Under the Precision-Recall Curve (AUC-PR) metrics. Please see Supplementary Figure S2 for a visual depiction of pathological classification performance attained by RF (gray boxplot), fully-connected networks (cyan boxplots), and PiDeeL (navy blue boxplots) with 2, 3, or 4 layers. Here, similar to the survival analysis results presented in Section 3.2 of the manuscript, we observe that PiDeeL consistently improves upon the fully-connected baseline, regardless of architecture depth or the performance metrics used for the current evaluation. In the case of AUC-ROC, we see 1.7%, 0.7%, and 2.7% improvement in performance PiDeeL compared with the fully-connected baseline with 2, 3, and 4 layers. As implicitly mentioned

above, we see a similar trend for the AUC-PR metric where improvements are 0.6%, 0.4%, and 0.8% for the same order of model depth. However, for the current setup of the pathological classification task, we interestingly do not quantitatively observe a big improvement with PiDeeL over the RF model. We observe that when the number of layers in fully-connected networks increases, the performance of the pathological classification deteriorates. However, for PiDeeL, this does not apply to the AUC-ROC metric where the median AUC-ROC slightly increases with the depth of the model.

1.4.2 Multi-task Learning

We explore a multi-task strategy for PiDeeL for survival analysis and pathological classification tasks. The multi-task PiDeeL learns a function $s : \mathbb{R}^{37} \rightarrow [-\infty, \infty] \times \{0, 1\}$. Note that we use the same feature vector (metabolite profiles) as input. The multi-task PiDeeL model architecture can be summarized as follows:

$$PL^{(138)} = \Big[\ FC^{(138)} weights\ \Big] \Big[PI\_matrix \Big] \tag{4}$$

$$PA^{(1:N)} = ReLU(PL^{(138)}(\widehat{M}^{(1:N)}) + b^{138}) \tag{5}$$

$$X_{hid}^{(1:N)} = ReLU(FC^{(64)}(PA^{(1:N)})) \tag{6}$$

$$\widehat{h}^{(1:N)}, \widehat{Y_m}^{(1:N)} = FC^{(2)}(X_{hid}^{(1:N)}) \tag{7}$$

We train 2,3 and 4-layer multi-task PiDeeL with the negative log of Cox Partial Likelihood and class-weighted binary cross-entropy losses. Additionally, hyperparameter configurations of multi-task PiDeeL were selected based on optimizing a linear combination of the survival and pathological classification surrogates. Specifically, a grid search based on validation performance led to a 0.125-fold weight for COX-PH loss and a 0.875-fold weight for binary cross-entropy loss. Please see Supplementary Figure S5 for a visualization of the convergence of the loss functions during training for a 3-layer PiDeeL model using a random seed. The left plot shows the absolute values of the Cox-PH and binary cross-entropy losses, while the right plot shows the scaled values of Cox-PH and binary cross-entropy losses.

Please see Supplementary Figure S3 for the performance comparison of single and multi-task learning of survival analysis and pathological classification tasks using PiDeeL. Across all analyzed model depths, we observe that the multi-task modeling strategy is at least slightly beneficial for performance on the survival analysis task. The 4-layer multitask PiDeeL attains the highest median c-index of 69.5%. We observe that the 2-layer multi-task PiDeeL outperforms all single-task and multi-task models in the pathological classification task with a median AUC-ROC of 91.9% and a median AUC-PR of 97.5%. Nonetheless, the examination of multi-task learning of PiDeeL yielded a slight improvement in performance (0.8% in median c-index and 1.8% in median AUC-ROC and 0.4% in median AUC-PR, in 4-layer and 2-layer multi-task PiDeeL models); thus, we decide to proceed with the utilization of two task-specific single-task PiDeeL models.

1.4.3 Interpretability and Importance Analyses

In this section, we cover the details of interpretability and importance analyses performed on PiDeeL for metabolite and pathway-activation vectors. First, we adopt the same 5-fold cross-validation fold setup (repeated 3 times) and hyperparameter selection described in Section 3.1 of

the manuscript. During iteration i, SHAP values are calculated on the test fold (fold$(i+1) mod 5$) on the trained PiDeeL model of training fold i. The hyperparameters are preserved among all trained models (5-fold repeated 3 times). In each iteration (i.e., 15 times), we concatenate the SHAP values calculated in this iteration with the previous SHAP values to get a $37x15$ matrix. We averaged each metabolite's SHAP value vector and sorted the 37 metabolite's mean SHAP values in increasing order. Please see Supplementary Figure S4 for the calculated SHAP values for each metabolite for survival analysis and pathological classification tasks. From this analysis, 3 metabolites with the biggest impact on PiDeeL's prediction on survival analysis are discussed in Section 3.3 of the manuscript.

After that, to assess the interpretability of the pathway profiles, we use the same 5-fold cross-validation setup. But this time, we take the first hidden layer's output (i.e., pathway-activation vector $PA^{(1:N)}$). We measure the impact of each pathway by calculating the SHAP values of vector $PA^{(1:N)}$ on PiDeeL's prediction. We again concatenate and sort SHAP values of $PA^{(1:N)}$) calculated on each fold. The 3 most important pathways are discussed in Section 3.3 of the manuscript.

Biological Motivation for Interpretability and Importance Analyses

Through a case-control study, alanine was reported to be a potential biomarker for malignant gliomas [11]. Similarly, glutamate was found to play a central role in malignant gliomas through multiple mechanisms [7]. Since malignant gliomas usually have a poor prognosis, the high ranking of alanine and glutamate in the list is expected. Furthermore, glutamine is the primary precursor of glutamate and plays a critical role in brain function [15]. Glutamine metabolism can regulate multiple pathways including energy production and redox homeostasis in brain cancer cells [15]. Glutamine itself was pinpointed as a candidate biomarker for glioma progression and response to treatment [9]. Thus, the rank of glutamine in the list is not surprising.

We biologically motivate the connection between the top 3 pathways and gliomas. Minerals and their absorption are fundamentally important to sustain life[1]. Calcium was reported to be one of the dietary components having a potential for glioma prevention by affecting apoptosis and DNA repair [20]. Thus, the rank of Mineral absorption pathway according to calculated importance is biologically grounded. Furthermore, two of the three metabolites implicated in Alanine, aspartate and glutamate metabolism pathway, were also detected among the top 3 important metabolites with respect to metabolite-level importance scores, as discussed above. Finally, mTOR is reported to have a key role in integrating signal transduction and metabolic pathways in glioblastoma [1]. Since $\sim 46\%$ of the cohort analyzed in this work were diagnosed with glioblastoma, the importance rank of the mTOR signaling pathway is viable. This qualitatively reinforces the predictive capabilities of PiDeeL and showcases its interpretability in both metabolite and pathway levels.

We also investigate the relationship between the top metabolites and pathways reported. Glutamine was reported to play a crucial role in the mTOR signaling pathway acting as a key nitrogen donor, activating mTORC1 [19]. mTORC1 regulates cell growth, metabolism, and autophagy, thus proliferation. Glutamine's activation effect on the mTORC1 complex controls protein synthesis, lipid synthesis, and autophagy which is usually altered in cancer metabolism [14]. Glutamate forms complexes with minerals through chelation. In this context, it can bind to minerals like calcium, sodium, and potassium. These complexes can enhance the solubility and availability of these minerals, making them more accessible to brain tissue [18]. Alanine acts as a glutamate-intake agent in

[1] KEGG PATHWAY: Mineral absorption - Homo sapiens (human). (n.d.). https://www.genome.jp/pathway/hsa04978

brain tissue through aminotransferase, which in turn regulates mineral absorption [10]. The alanine, aspartate, and glutamate metabolism pathway is responsible for the synthesis and interconversion of amino acids. It plays a crucial role in the synthesis of arginine, asparagine, aspartate, alanine, glutamate, glutamine, and proline[2]. It's also important to emphasize that the alanine, aspartate, and glutamate metabolism pathway is highly connected with other metabolic pathways. That is, glutamate donates amino groups for the synthesis of other amino acids, such as non-essential amino acids[3] [5]. Glutamate can be converted into alpha-ketoglutarate through the action of the enzyme glutamate dehydrogenase[2]. This reaction generates reducing equivalents (NADH) and participates in the tricarboxylic acid (TCA) cycle, a key pathway for energy production[4]. Glutamate is a precursor for glutathione, a critical antioxidant molecule that helps protect cells from oxidative damage and maintain redox balance [12].

1.4.4 Ablation Studies

In this section, we give the details of the ablation tests we performed to identify the contribution of the pathway-informed architecture to the performance of the PiDeeL model.

In test (a), we validate the contribution of the number of neurons in the first layer to the performance. Note that in PiDeeL, the first hidden layer has 138 neurons, whereas trained fully-connected networks (baseline models) have 64. We train 2, 3, and 4-layer DeepSurv with the same 5-fold cross-validation setup, and repeat it 3 times. The model architecture can be summarized as follows:

$$X_{hid}^{(1:N)} = ReLU(FC^{(138)}(\widehat{M}^{(1:N)})) \tag{8}$$

$$X_{hid}^{(1:N)} = ReLU(FC^{(64)}(X_{hid}^{(1:N)})) \tag{9}$$

$$\widehat{h}^{(1:N)} = FC^{(1)}(X_{hid}^{(1:N)}) \tag{10}$$

Second, in test (b), we validate the contribution of the first hidden layer's sparsity to the performance of PiDeeL. The total connections between the input layer ($\widehat{M}^{(1:N)}$) and the first hidden layer $PL^{(1:N)}$ are 468 in PiDeeL, whereas in a fully-connected network with 37 input features, there are 5106 (considering the case of 138 first hidden layer neurons). We train 2, 3, and 4-layer DeepSurv with dropout in the first hidden layer adopting the same 5-fold cross-validation setup, and repeat it 3 times. We train fully-connected networks using different dropout rates from $\{0.5, 0.6, 0.7, 0.8, 0.9\}$.

Recall that $PL^{(138)}$ refers to the weights of the pathway-informed layer of PiDeeL. To construct the $PL^{(138)}$, we use Eq 8 where we multiply $FC^{(138)}weights$ with PI_matrix. Note that we build PI_matrix from the KEGG database for pathways. In test (c), we use randomly connected PI_matrix (to verify the contribution of PI_matrix to the performance of PiDeeL. The number of connections in the first hidden layer (468) is preserved in random PI_matrix.

$$PL^{(138)} = \left[\ FC^{(138)}weights\ \right]\left[PI\_matrix\right] \tag{11}$$

[2]KEGG PATHWAY: Alanine, aspartate and glutamate metabolism - Reference pathway. (n.d.). https://www.genome.jp/pathway/map00250

[3]KEGG PATHWAY: Nitrogen metabolism - Reference pathway. (n.d.). https://www.genome.jp/pathway/map00910

[4]KEGG PATHWAY: Citrate cycle (TCA cycle) - Reference pathway. (n.d.). https://www.genome.jp/pathway/map00020

We train 2, 3, and 4-layer PiDeeL with the same 5-fold cross-validation setup, and repeat it 30 times (i.e., 150 iterations).

In test (d), we construct PI_matrix similar to test (c). However, we shuffle each metabolite-to-pathway connection vector instead of randomly connecting. Note that PI_matrix is a $37x138$ matrix; and for metabolite j, $PI\_matrix.j$ yields a vector of shape $1x138$. We shuffle these 37 vectors and train 2, 3, and 4-layer PiDeeL with the same 5-fold cross-validation setup, and repeat it 30 times (i.e., 150 iterations).

Finally, in test (e), we train and test DeepSurv and PiDeeL on sample sets of sizes 50, 100, and 200. We randomly undersample our dataset (n=384) to sizes 200, 100, and 50. We then train and test DeepSurv and PiDeeL on these sample sets for 10 times with a 5-fold cross-validation and repeat it 3 times. Note that this means 150 iterations in total.

Please see Figures 3 and 4 in the manuscript for the depiction of the results of ablation studies. Please refer to Section 3.4 of the manuscript for the observations of ablation tests for the survival analysis task.

1.4.5 Full spectrum model

We consider a fully-connected MLP model that takes the full spectrum as input. Note that this model excludes the automated metabolite quantification workflow discussed in Section 1.2 of the Supplementary Material. We train and test 2, 3, and 4-layer versions of this model with the same strategy we use to train and test PiDeeL. Also, note that the model architecture is the same as the baseline fully-connected models we use except for the shape of the input vector (16,314 instead of 37).

Please see Figure S6 of the Supplementary Material for the performance comparison of these models and PiDeeL in terms of the c-index. PiDeeL improves the performance attained by DeepSurv using the whole spectrum as input in terms of median c-index by 7.9%, 8.3%, and 5.0% for 2, 3, and 4-layered networks, respectively. Additionally, PiDeeL decreases the variance by an average of 21.0%.

1.4.6 PiDeeL with different losses

We train a PiDeeL using losses formulated by DeepHit, PC-Hazard, and DeepSurv models in Eq 9, 12, and 13 of the manuscript. Given our dataset of N samples, the architecture of the trained 4-layer PiDeeL model for the survival analysis can be summarized as follows:

$$PL^{(138)} = \Big[\ FC^{(138)} weights\ \Big] \Big[PI\_matrix \Big] \tag{12}$$

$$PA^{(1:N)} = ReLU(PL^{(138)}(\widehat{M}^{(1:N)}) + b^{138}) \tag{13}$$

$$X_{hid}^{(1:N)} = ReLU(FC^{(64)}(PA^{(1:N)})) \tag{14}$$

$$X_{hid}^{(1:N)} = ReLU(FC^{(64)}(PA^{(1:N)})) \tag{15}$$

$$\widehat{h}^{(1:N)} = FC^{(1)}(X_{hid}^{(1:N)}) \tag{16}$$

We train 4-layer PiDeeL with the same 5-fold cross-validation setup and repeat it 30 times (i.e., 150 iterations). In Supplementary Figure S8, we show the performance comparison of PiDeeL using DeepHit, PC-Hazard (cyan boxplots), and DeepSurv (navy blue boxplot) losses. PiDeeL trained with DeepSurv loss outperformed PiDeeL models trained with other losses in the median c-index

metric. In particular, using DeepSurv loss improved the median c-index attained by using DeepHit and PC-Hazard losses by 9.75% and 4.04%. Also, DeepSurv loss decreased the variance by 22.1% and 39.2% for DeepHit and PC-Hazard losses, respectively. Thus, we proceed with using DeepSurv loss formulated as given in Eq 13 of the manuscript in our model.

1.4.7 PiDeeL and other models (DeepHit, PC-Hazard, and DeepSurv)

We consider 2, 3, and 4-layer DeepHit, PC-Hazard, and DeepSurv models as baseline models. For DeepSurv, we use a single neuron in the last layer with linear activation to output the predicted risk scores of samples. For DeepHit and PC-Hazard models, the number of neurons in the last layer is a tuneable parameter, named *num_durations*. We empirically select 10 as *num_durations* among $\{5, 10, 20, 50, 100\}$.

The trained 3-layer DeepHit and PC-Hazard models on our dataset of size N can be summarized as follows:

$$X_{hid}^{(1:N)} = ReLU(FC^{(64)}(\widehat{M}^{(1:N)})) \tag{17}$$

$$X_{hid}^{(1:N)} = ReLU(FC^{(64)}(X_{hid}^{(1:N)})) \tag{18}$$

$$\widehat{h}^{(1:N)} = FC^{(10)}(X_{hid}^{(1:N)}) \tag{19}$$

The trained 3-layer DeepSurv model on our dataset of size N can be summarized as follows:

$$X_{hid}^{(1:N)} = ReLU(FC^{(64)}(\widehat{M}^{(1:N)})) \tag{20}$$

$$X_{hid}^{(1:N)} = ReLU(FC^{(64)}(X_{hid}^{(1:N)})) \tag{21}$$

$$\widehat{h}^{(1:N)} = FC^{(1)}(X_{hid}^{(1:N)}) \tag{22}$$

where X_{hid} represents the output of the first hidden layer, $\widehat{h}^{(i)}$ represents the predicted risk score, $FC^{(\cdot)}$ represents a fully-connected layer with $(\cdot)$ neurons, and $ReLU$ represents the rectified linear unit. In the case of 2 and 4-layer DeepHit, PC-Hazard, and DeepSurv models, we remove or repeat the hidden layer defined in Eq 18 and 21.

Please see Supplementary Figures S9, S10, and S11 for layerwise comparisons between PiDeeL and DeepHit, PC-Hazard and DeepSurv. We see that the performance slightly increases when the depth of PiDeeL increases from 2 to 4 (improvement up to median c-index of 0.4%). On the other hand, the performance of PC-Hazard and DeepSurv models diminishes with increasing model depth (deterioration up to a median c-index of 4.4% and 1.5% for PC-Hazard and DeepSurv, respectively). Furthermore, when neural network-based models (DeepHit, PC-Hazard, and DeepSurv) and PiDeeL with the same number of layers are compared, we see the performance gap increase with respect to the number of layers in the model. Here, we quantitatively observe the benefit of incorporating biological information in modeling choices. For 2, 3, and 4-layered networks, PiDeeL improves upon DeepHit with the same model depth by 15.8%, 13.1%, and 12.0% according to the median c-index, respectively. PiDeeL improves upon PC-Hazard with the same model depth by 2.6%, 4.0%, and 7.9% according to the median c-index. Finally, PiDeeL improves upon DeepSurv with the same model depth by 2.5%, 3.4%, and 4.3% according to the median c-index. These support the robustness of sparse architectures in small data regimes for the survival analysis task.

1.4.8 Data Simulation

We obtained an independent HRMAS NMR glioma dataset from (Firdous et al., 2021) [11]. We preprocessed raw FID files as we do for our dataset. The result of the preprocessing pipeline is a 16.314-long spectrum vector. We then normalized the signal intensity with respect to the mean intensity in our samples. We shifted the spectrum to the left by 1515 ppm for calibration to align with our samples. This dataset consists of 42 individuals, 26 of which are glioma samples. First, we predict the metabolite quantities for these 26 samples to obtain the feature vectors.

The labels in the Firdous et al. dataset consist of tumor grade (i.e., I - IV) and tumor subtype (e.g. AST, GBM). Our dataset also contains these two labels, namely tumor grade and tumor subtype. As the Firdous et al. dataset lacks the survival information (i.e., event indicator Y_e and/or time-to-event Y_d), we simulate $Y_e^{(i)}$ and $Y_d^{(i)}$ for each glioma sample (i), considering their grades and subtypes.

For each subtype and grade (e.g. ASTIII, OASTII), we assume that the survival labels of samples in the Firdous et al. dataset are uniformly distributed, with the minimum and maximum time-to-event variables in our dataset that corresponds to the same subtype and grade. Then, for each glioma sample in the Firdous et al. dataset, we randomly drew a time-to-event label from the corresponding distribution. Similarly, we assume that the event indicator in the Firdous et al. dataset follows a Bernoulli distribution for each subtype and grade such that the fraction of deceased and censored samples in that group are the same as in our dataset. We again drew randomly from the corresponding distribution to simulate an event indicator label. We repeated this simulation 100 times with 3 different random seeds (i.e., 300 total iterations).

We compared the survival prediction performance of other neural network-based models and PiDeeL on this simulated data. Please see Figure 6 (also given below) of the manuscript for the performance benchmark of these models and PiDeeL. Median c-indices of baseline models are under 50%, specifically, DeepHit, PC-Hazard, and DeepSurv attain a median c-index of 47.4%, 47.7%, and 46.9%, respectively. PiDeeL outperforms DeepHit, PC-Hazard, and DeepSurv models with a median c-index of 52.1%. Also, PiDeeL decreases the variance by an average of 15.2%.

2 Supplementary Figures

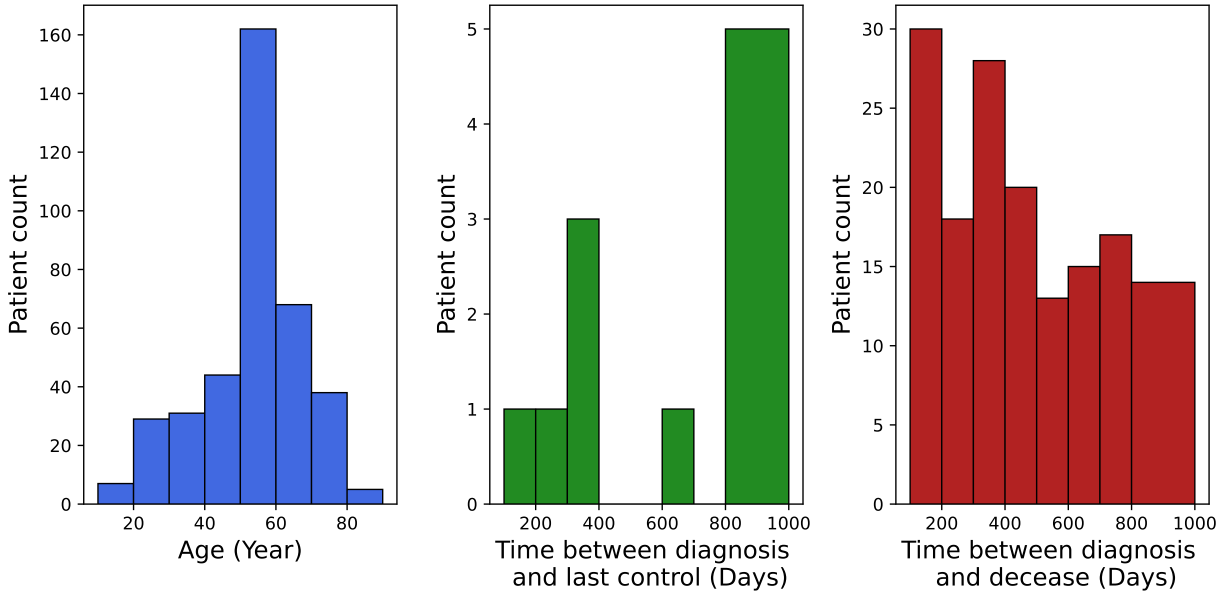

Supplementary Figure 1: (i) the age distribution of patients in the dataset, (ii) the distribution of duration between the primary tumor removal surgery and the last control, (iii) the duration distribution of duration between the primary tumor removal surgery and decease

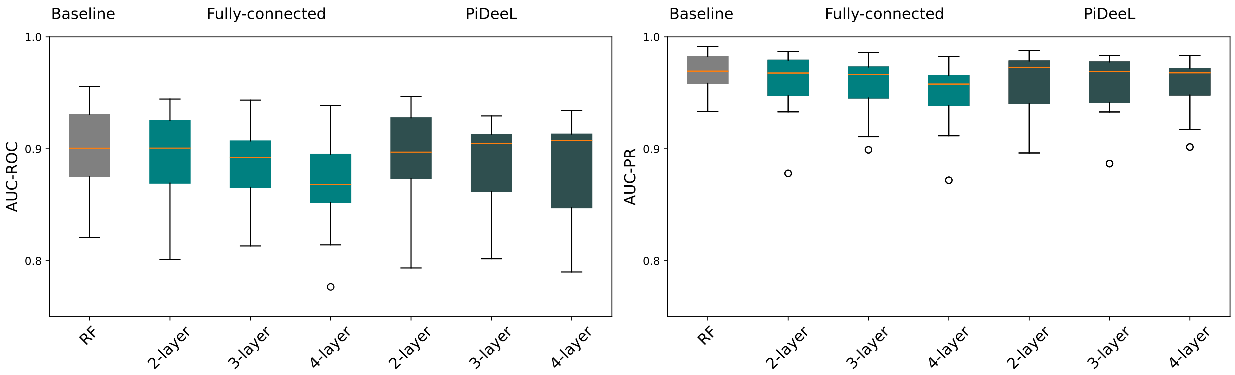

Supplementary Figure 2: Comparison of baseline models and PiDeeL on pathologic classification in terms of AUC-ROC (left) and AUC-PR (right). This evaluation is based on 5-fold cross-validation that is repeated 3 times. (i.e., 15 iterations)

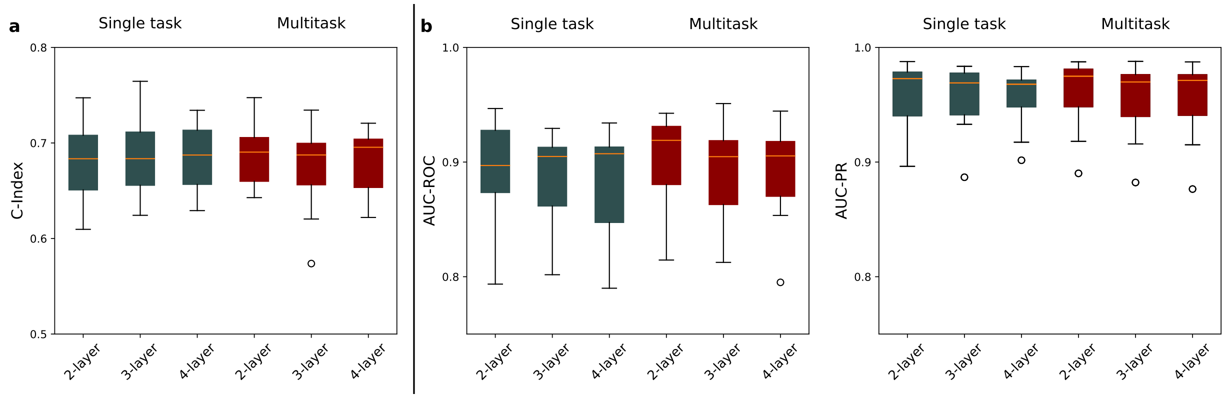

Supplementary Figure 3: The performance comparison between task-specific PiDeeL (navy blue boxplots) versus multi-task learning of PiDeeL (red boxplots). The survival analysis performance is under Panel A and the pathological classification performance is under Panel B, with AUC-ROC on the left, and AUC-PR on the right. This evaluation is based on 5-fold cross-validation that is repeated 3 times. (i.e., 15 iterations)

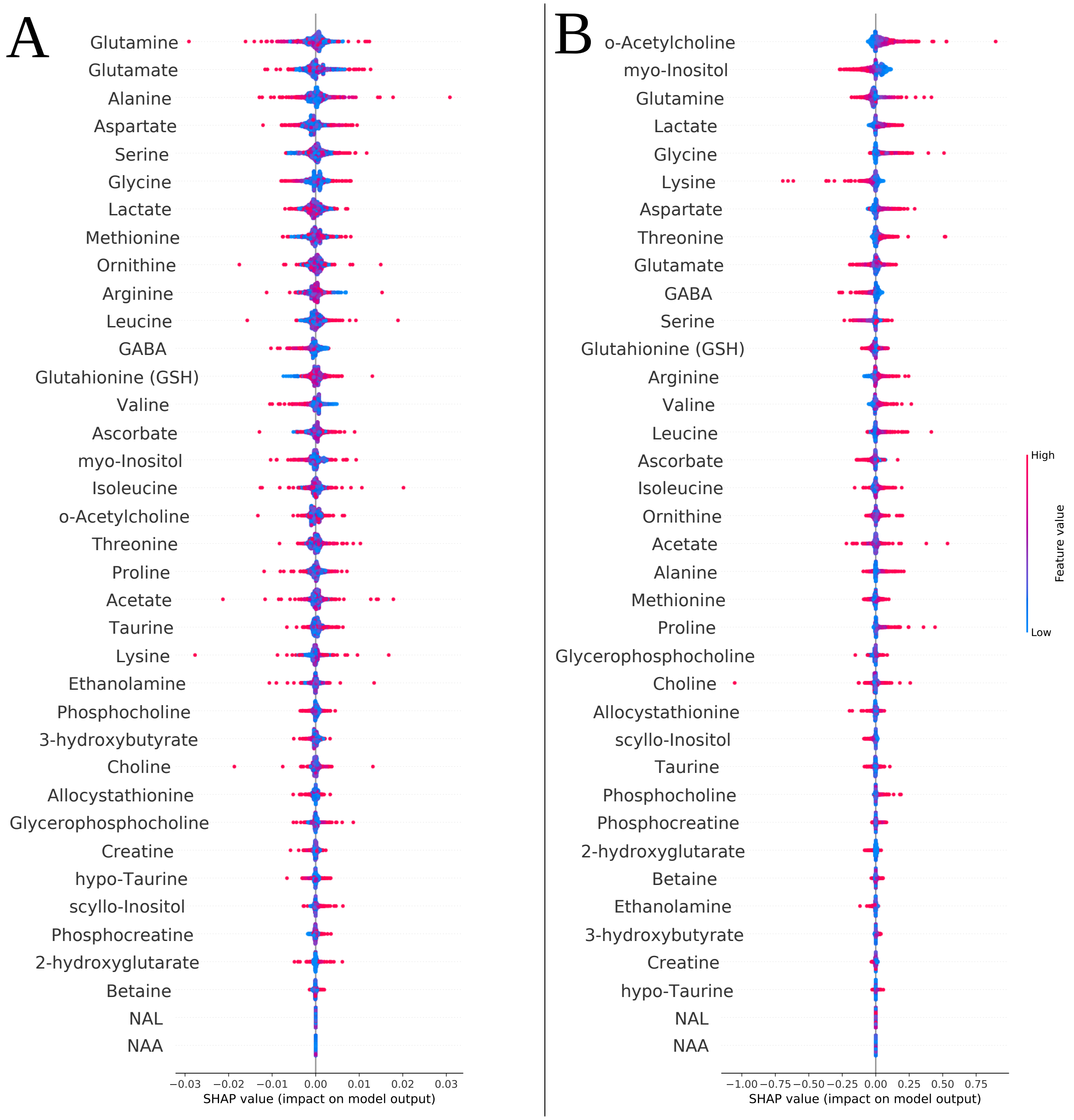

Supplementary Figure 4: Under Panel A of the figure, the SHAP values (on the x-axis) for the survival analysis task of each quantified biomarker metabolite (on the y-axis) are shown. Under Panel B of the figure, the SHAP values (on the x-axis) for the pathological classification task of each quantified biomarker metabolite (on the y-axis) are shown. The color bar is shown (red represents a higher value and blue represents a lower value for a feature). The more the SHAP value, the more critical the metabolite is for the model's output toward a higher risk score for the survival analysis task and a high probability of malignant tumor for the pathological classification task.

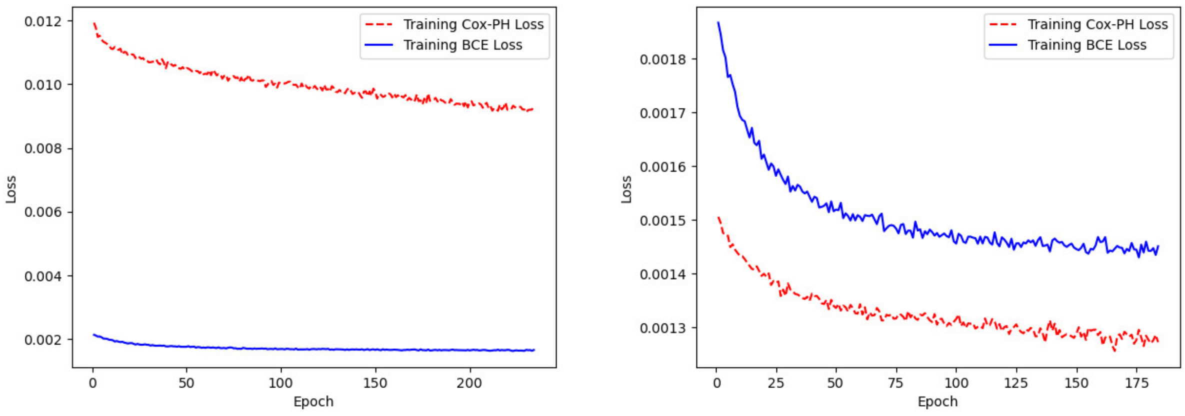

Supplementary Figure 5: The training loss convergence during training for the 3-layer PiDeeL model using a random seed. On the left, the absolute values of Cox-PH and BCE losses are shown, whereas, on the right, the scaled loss values are shown. The weights for Cox-PH and BCE losses are 0.125 and 0.875, respectively.

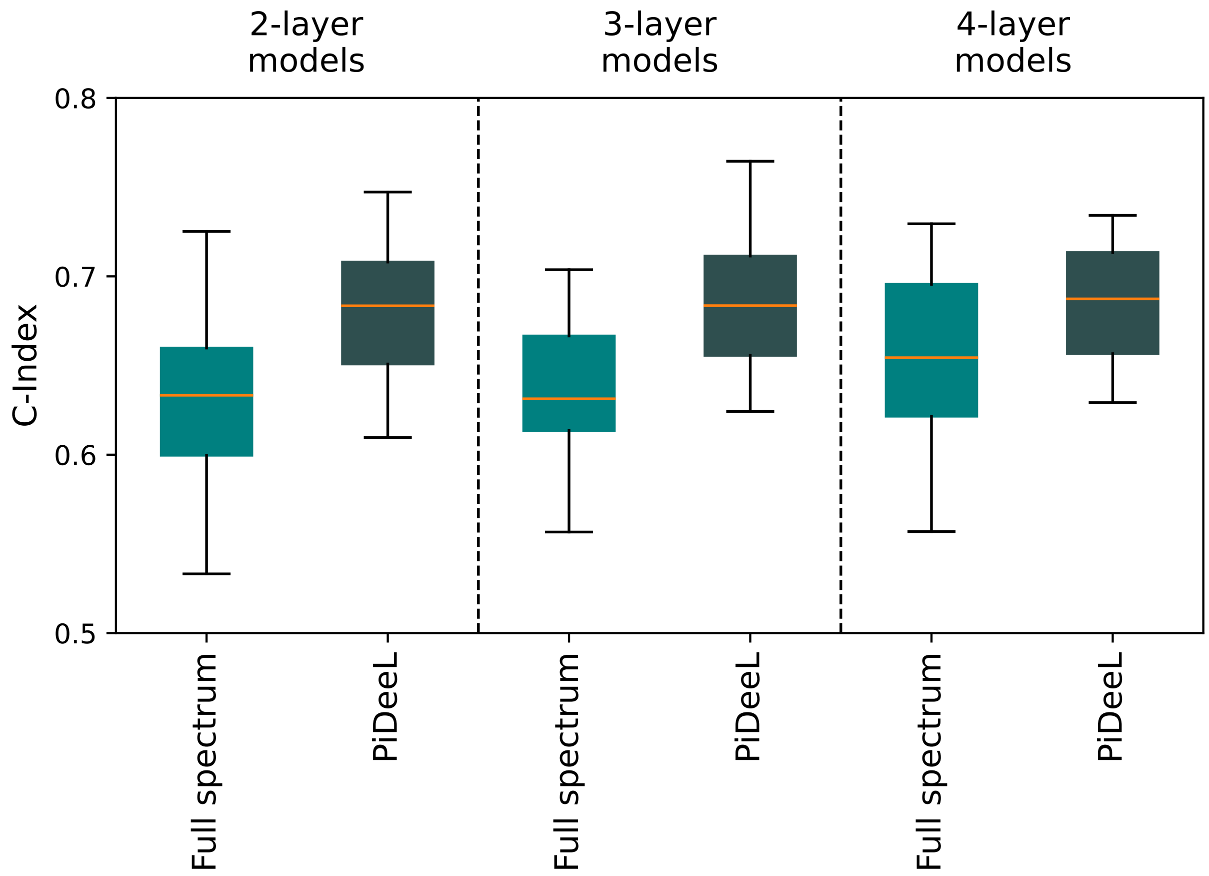

Supplementary Figure 6: Performance comparison of DeepSurv using full spectrum input and PiDeeL on survival analysis in terms of the c-index. This evaluation is based on a 5-fold cross-validation that is repeated 3 times. (i.e., 15 iterations)

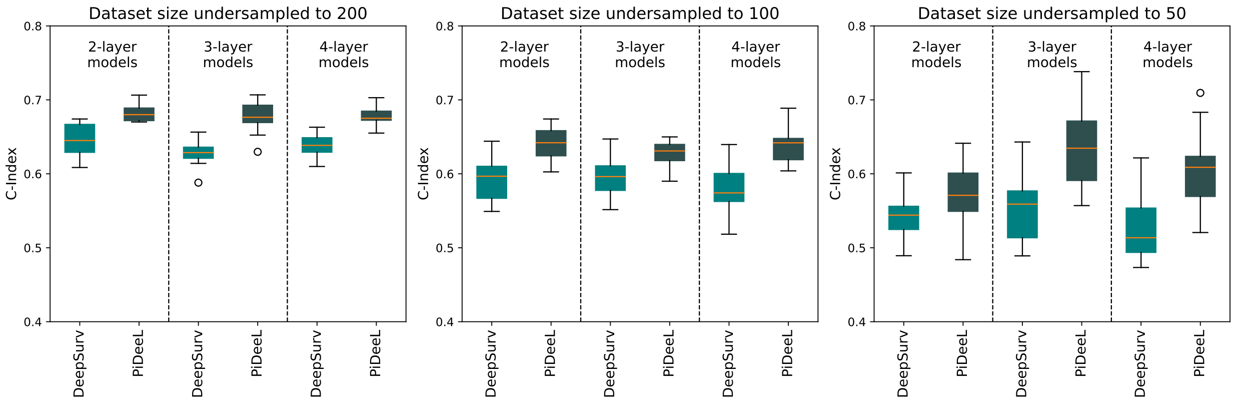

Supplementary Figure 7: Performance comparison of DeepSurv and PiDeeL on survival analysis in terms of the c-index on different sizes of datasets. This evaluation is based on a 5-fold cross-validation that is repeated 3 times. (i.e., 15 iterations)

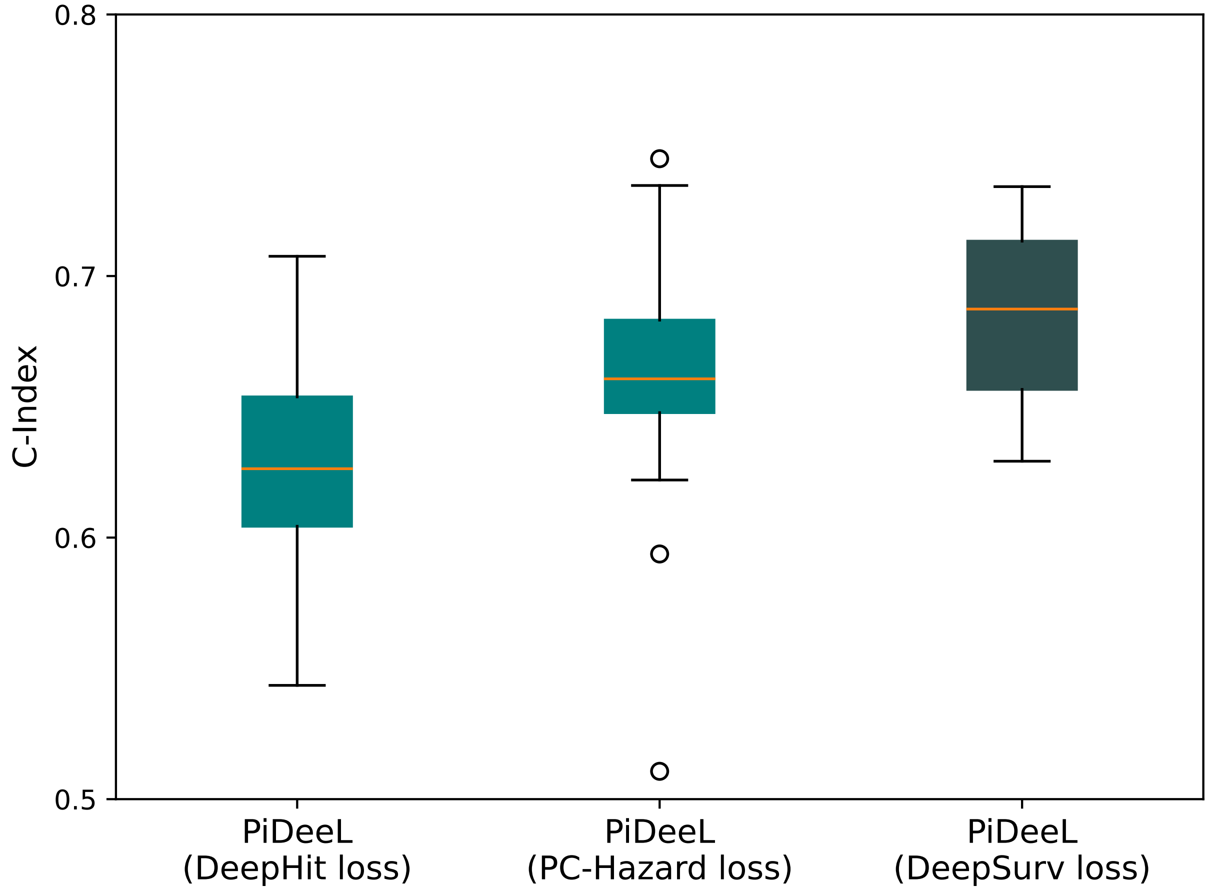

Supplementary Figure 8: Performance comparison of PiDeeL with different losses on survival analysis in terms of the c-index. This evaluation is based on a 5-fold cross-validation that is repeated 3 times. (i.e., 15 iterations)

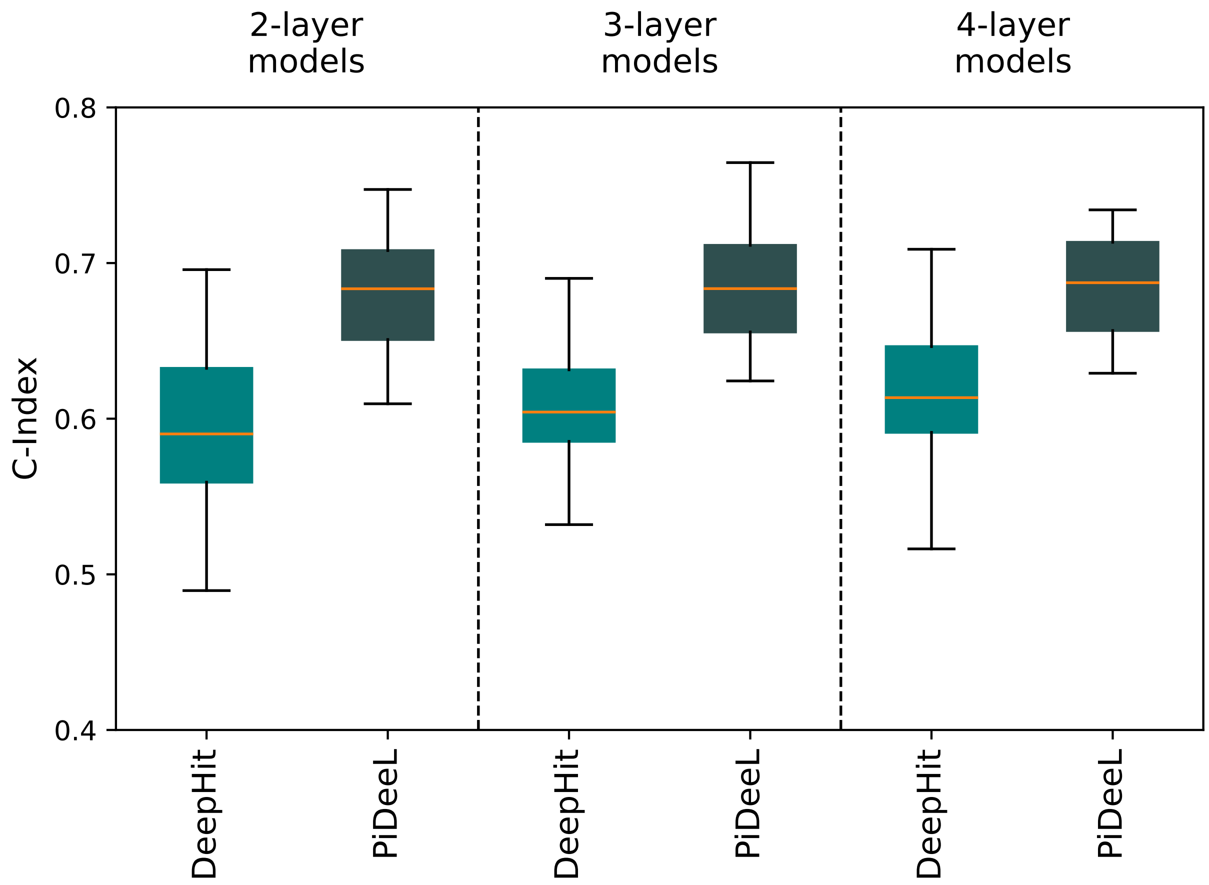

Supplementary Figure 9: Performance comparison of DeepHit and PiDeeL on survival analysis in terms of the c-index. This evaluation is based on a 5-fold cross-validation that is repeated 3 times. (i.e., 15 iterations)

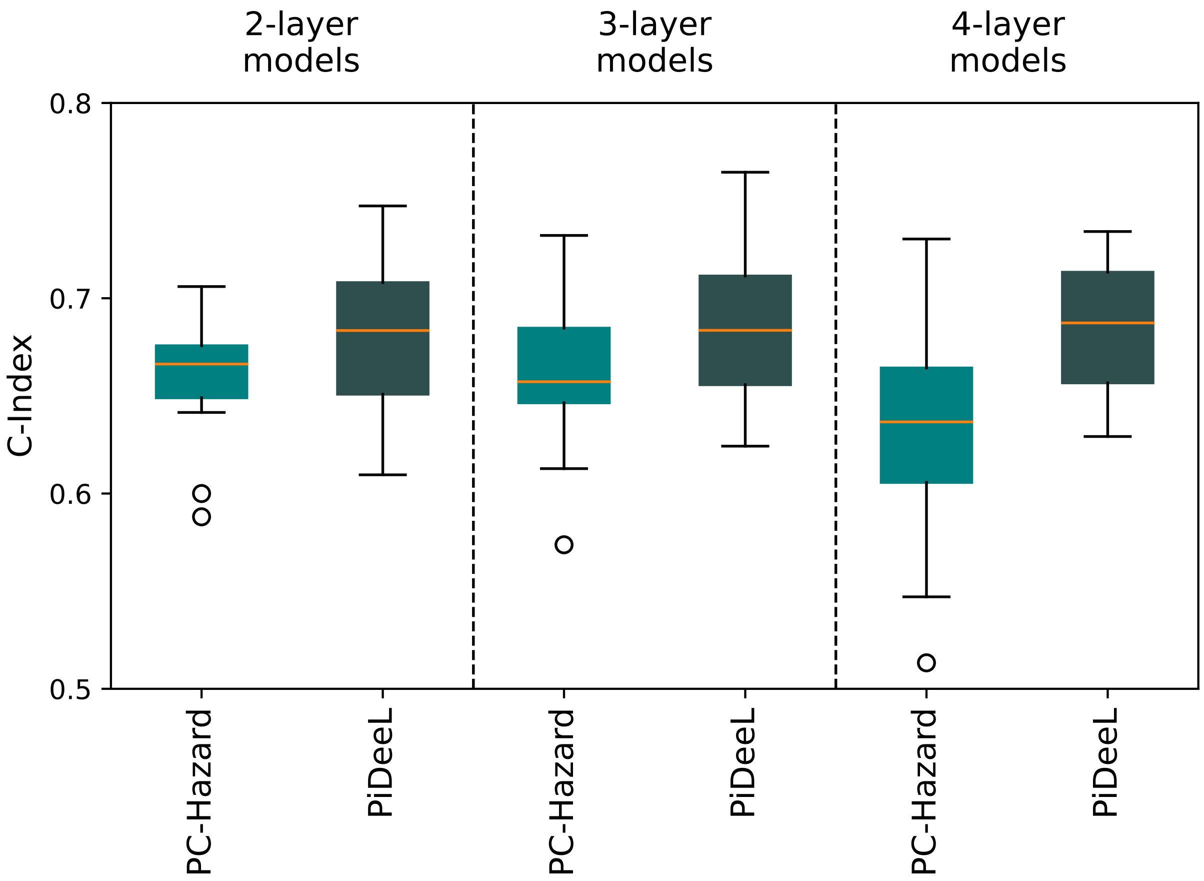

Supplementary Figure 10: Performance comparison of PC-Hazard and PiDeeL on survival analysis in terms of the c-index. This evaluation is based on a 5-fold cross-validation that is repeated 3 times. (i.e., 15 iterations)

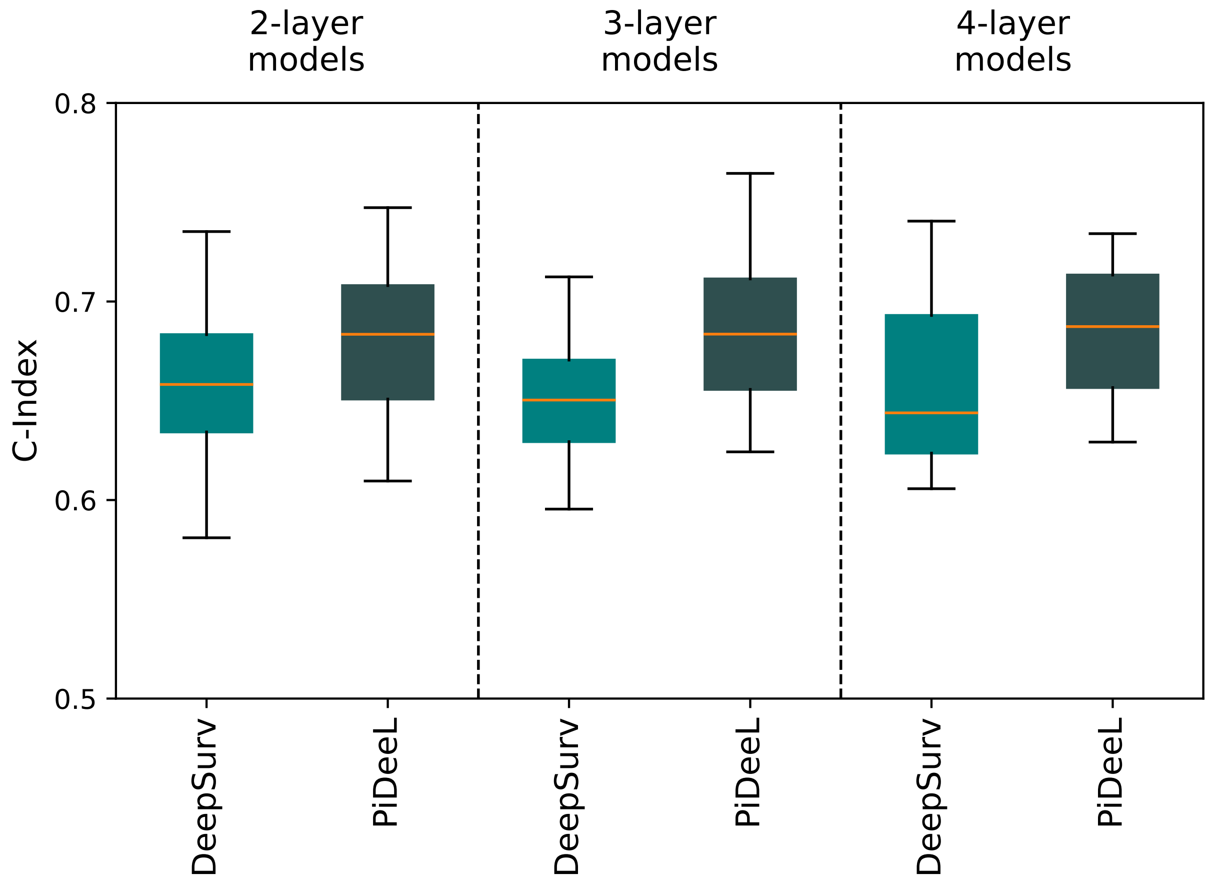

Supplementary Figure 11: Performance comparison of DeepSurv and PiDeeL on survival analysis in terms of the c-index. This evaluation is based on a 5-fold cross-validation that is repeated 3 times. (i.e., 15 iterations)

3 Supplementary Tables

Primary Brain Tumor Types	Number of Samples
Pilocytic astrocytoma (AST-I)	4
Astrocytoma grade II (AST-II)	5
Astrocytoma grade III (AST-III)	7
Glioblastoma (GBM)	178
Oligodendroglioma grade II (ODG-II)	36
Oligodendroglioma grade III[1] (ODG-III)	103
Oligoastrocytoma grade II (OAST-II)	3
Oligoastrocytoma grade III (OAST-III)	9
Ganglioma grade II (GG-II)	5
Ganglioma grade III (GG-III)	1
Dysembryoplastic Neuroepithelial Tumors (DNET)	25
Gliosarcoma (GS)	3
Unkown subtype	5

Supplementary Table 1: Histopathological classification of the tumor tissue specimens used in the study.

[1]Three samples had glioblastoma as well as oligodendroglioma grade III. They are included in the sample count for the latter.

References

[1] David Akhavan, Timothy F Cloughesy, and Paul S Mischel. mtor signaling in glioblastoma: lessons learned from bench to bedside. *Neuro-oncology*, 12(8):882–889, 2010.

[2] Serge Akoka, Laurent Barantin, and Michel Trierweiler. Concentration measurement by proton nmr using the eretic method. *Analytical Chemistry*, 71(13):2554–2557, 1999.

[3] Laura Antolini, Patrizia Boracchi, and Elia Biganzoli. A time-dependent discrimination index for survival data. *Statistics in medicine*, 24(24):3927–3944, 2005.

[4] Norman Breslow. Covariance analysis of censored survival data. *Biometrics*, pages 89–99, 1974.

[5] John T Brosnan and Margaret E Brosnan. Glutamate: a truly functional amino acid. *Amino acids*, 45:413–418, 2013.

[6] Doruk Cakmakci, Gun Kaynar, Caroline Bund, Martial Piotto, Francois Proust, Izzie Jacques Namer, and A Ercument Cicek. Targeted metabolomics analyses for brain tumor margin assessment during surgery. *Bioinformatics*, 2022.

[7] John de Groot and Harald Sontheimer. Glutamate and the biology of gliomas. *Glia*, 59(8):1181–1189, 2011.

[8] Bradley Efron. The efficiency of cox's likelihood function for censored data. *Journal of the American statistical Association*, 72(359):557–565, 1977.

[9] Selin Ekici, Benjamin B Risk, Stewart G Neill, Hui-Kuo Shu, and Candace C Fleischer. Characterization of dysregulated glutamine metabolism in human glioma tissue with 1h nmr. *Scientific reports*, 10(1):1–10, 2020.

[10] Maria Ereciska, David Nelson, Itzhak Nissim, Yevgeny Daikhin, and Marc Yudkoff. Cerebral alanine transport and alanine aminotransferase reaction: alanine as a source of neuronal glutamate. *Journal of neurochemistry*, 62(5):1953–1964, 1994.

[11] Safia Firdous, Rizwan Abid, Zubair Nawaz, Faisal Bukhari, Ammar Anwer, Leo L Cheng, and Saima Sadaf. Dysregulated alanine as a potential predictive marker of glioma—an insight from untargeted hrmas-nmr and machine learning data. *Metabolites*, 11(8):507, 2021.

[12] S.C. Gad. Glutathione. In Philip Wexler, editor, *Encyclopedia of Toxicology (Third Edition)*, page 751. Academic Press, Oxford, third edition edition, 2014.

[13] Diederik P Kingma and Jimmy Ba. Adam: A method for stochastic optimization. *arXiv preprint arXiv:1412.6980*, 2014.

[14] Delong Meng, Qianmei Yang, Huanyu Wang, Chase H Melick, Rishika Navlani, Anderson R Frank, and Jenna L Jewell. Glutamine and asparagine activate mtorc1 independently of rag gtpases. *Journal of Biological Chemistry*, 295(10):2890–2899, 2020.

[15] Siva Kumar Natarajan and Sriram Venneti. Glutamine metabolism in brain tumors. *Cancers*, 11(11):1628, 2019.

[16] Adam Paszke, Sam Gross, Francisco Massa, Adam Lerer, James Bradbury, Gregory Chanan, Trevor Killeen, Zeming Lin, Natalia Gimelshein, Luca Antiga, et al. Pytorch: An imperative style, high-performance deep learning library. *Advances in neural information processing systems*, 32, 2019.

[17] Sebastian Pölsterl. scikit-survival: A library for time-to-event analysis built on top of scikit-learn. *Journal of Machine Learning Research*, 21(212):1–6, 2020.

[18] I Pull, H McIlwain, and RL Ramsay. Glutamate, calcium ion-chelating agents and the sodium and potassium ion contents of tissues from the brain. *Biochemical Journal*, 116(2):181–187, 1970.

[19] Hayden Weng Siong Tan, Arthur Yi Loong Sim, and Yun Chau Long. Glutamine metabolism regulates autophagy-dependent mtorc1 reactivation during amino acid starvation. *Nature communications*, 8(1):338, 2017.

[20] Nicole Tedeschi-Blok, Judith Schwartzbaum, Marion Lee, Rei Miike, and Margaret Wrensch. Dietary calcium consumption and astrocytic glioma: the san francisco bay area adult glioma study, 1991-1995. *Nutrition and cancer*, 39(2):196–203, 2001.

[21] Gerhard Wider and Lars Dreier. Measuring protein concentrations by nmr spectroscopy. *Journal of the American Chemical Society*, 128(8):2571–2576, 2006.
